# In masks we trust: explicit and implicit reactions to masked faces vary by political orientation

**DOI:** 10.1186/s40359-024-01556-5

**Published:** 2024-02-12

**Authors:** Gordon P. D. Ingram, Erick G. Chuquichambi, William Jimenez-Leal, Antonio Olivera-La Rosa

**Affiliations:** 1 Departamento de Psicología, Universidad de los Andes, Carrera 1 # 18A-12, 111711 Bogotá, Colombia; 2 Human Cognition and Evolution (EvoCog) Research Group, University of the Balearic Islands, Palma, Spain; 3 Universidad de los Andes, Bogotá, Colombia; 4 Universidad Católica Luis Amigó, Medellín, Colombia

**Keywords:** COVID-19, Face perception, Moralization, Reaction times, Social distance, Trust

## Abstract

Previous studies in the context of the COVID-19 pandemic indicated that wearing a medical-style mask affects whether a stranger’s face is judged as more trustworthy, socially desirable, or likely to be ill. However, given political controversies around mask use, these effects might vary by political orientation. In a pre-registered online experiment, we measured evaluations of trustworthiness, social desirability and perceived illness in masked and unmasked faces by 1241 British and US participants. We included questions on political orientation, along with the implicit online-VAAST approach/avoid task to test reaction times to masked/unmasked faces. There was a medium-sized effect of masks on trustworthiness and a significant interaction with political orientation, in that conservatives found masked faces less trustworthy than did liberals. Participants were quicker to approach masked than unmasked faces, but conservatives were relatively slower than liberals. The effects on trustworthiness suggest that differential moralization of novel social norms can affect how their adherents are evaluated in terms of their suitability for social interactions. Furthermore, the congruence between implicit and explicit methods implies that such differences can have deep-seated effects on reactions.

**Supplementary Information:**

The online version contains supplementary material available at 10.1186/s40359-024-01556-5.

During the COVID-19 pandemic, the wearing of medical-style face masks became ubiquitous in public places in many countries [1, 2]. However, substantial differences have existed between individuals and countries in the extent of mask adoption, and some countries have seen opposition to imposing the practice by law [2–4], which has often correlated with partisan political differences, especially in the USA [5] and UK. Besides the political context, individual differences in attitudes to mask-wearing are likely driven by a complex array of factors, including explicit judgements about health risks, an automatic tendency to follow social norms [6], and implicit reactions to covered faces. This last point is interesting because the social importance of face coverings, e.g., in rituals, dramatic performances, or anonymous acts of violence, has been discussed in theoretical terms by philosophers, anthropologists, and social psychologists [7–9]. Yet there are few empirical studies from before the pandemic that directly addressed the question of how people react to masked faces (but see [10, 11]).

Nevertheless, a theoretical case can be made for the relevance of masks to evaluations of the wearers’ trustworthiness. Visibility of facial expressions is important for processing information about feelings and intentions [12]: obscuring these could thus lead to appraisals of ambiguity, uncanniness, and potential social danger, which might decrease trust in the wearer [13, 14]. Such appraisals are likely automatic, since when viewing unfamiliar faces, we make automatic judgements of trustworthiness or untrustworthiness in literally a fraction of a second [12, 15]. Faces rated less trustworthy cause greater activity in the amygdala [16, 17], a brain area associated with spontaneous emotional evaluations (including fear), supporting the idea that people automatically evaluate unfamiliar faces for trustworthiness, with untrustworthy faces generating negative emotional responses. Hence, masking one’s face could affect observers’ automatic reactions, independently of their explicit attitudes to mask-wearing. Evaluations of trustworthiness might also co-vary with individual differences in generalized social trust [18]. Individuals high in such trust tend to assume that people have good intentions and lack malice, facilitating a predisposition towards social contact, even with strangers [19].

On the other hand, due to their association first with medical contexts and now the pandemic, masks might also be linked in people’s minds with sickness and disease. Theories of the “behavioral immune system” suggest we have evolved to avoid potentially contagious agents or substances [20]. This extends to face perception, with intuitive judgements of disease perhaps motivating the negative social evaluations that tend to be made of ugly or uncanny faces [13, 21, 22]. During a pandemic in which there is a high risk of infection from strangers, the desired level of social closeness with a stranger could easily be affected by the use of facial information to make intuitive judgments about their possible state of health. Wearing a medical-style mask might influence the perceived sickness of, and risk of contagion from, the wearer [23]. Perception of contagion risk could co-vary with disgust sensitivity: the ease and intensity with which individuals experience disgust [24, 25]. Disgust sensitivity may be related to certain psychiatric disorders [26], personality traits [27], and social prejudices [28]. Fan and Olatunji [29] showed that individual differences in disgust predicted "health-related anxiety" and the avoidance of stimuli associated with the common cold – thus, they could also conceivably lead to avoidance of masked individuals.

Although there was a lack of empirical research into the impact of masks on face perception before the pandemic, a study in the first half of 2020 addressed the perceived trustworthiness and sickness of mask-wearers through an online experiment with a Spanish-speaking sample [23]. Two groups of participants viewed a series of five faces, which either were not wearing masks, or were photoshopped with a basic surgical-style mask. After viewing each face, participants were asked to rate it on preferred social distance, trustworthiness, and sickness perception. Mask-wearers were perceived to be more trustworthy and socially desirable, yet also more likely to be ill, regardless of the levels of generalized social trust, social anxiety and disgust sensitivity that participants reported. The authors interpreted these results in the light of a new, “moralized” [30] social norm of mask-wearing that generated affiliative reactions in observers, causing increased evaluations of trustworthiness and a willingness to reduce social distance.

However, Olivera-La Rosa et al. also noted that masks had been quickly and widely adopted in the Spanish-speaking countries (principally Colombia) where they collected data. Therefore, these results might not generalize to other countries whose governments did not rapidly and widely mandate mask-wearing, contributing to the practice being less normative—more politically contested—in their populations [4]. Indeed, subsequent studies in other countries found somewhat conflicting results, with masks resulting in a reduction in desired interpersonal distance among French participants [31], but decreasing the perceived social closeness of faces for a German sample [32]. Masking faces may have other effects on social perception as well, for example by impairing recognition of familiar and unfamiliar faces in both adults and children [33–35]; reducing the ability to accurately perceive and mimic emotional expressions [32, 36–38]; or even improving facial attractiveness when compared with the ratings given to masked faces in the pre-pandemic context, when masks seem to have been more strongly associated with unhealthiness [11, 39].

The diversity of findings on the perceptual effects of masks points to the potential role of moderator variables in affecting how different individuals react to them. In many countries (e.g., the USA and UK) whether an individual responds positively or negatively to face-masks seems likely to be affected by their degree of political conservatism or liberalism, in common with reactions to many other social practices that have become morally and politically charged [40]. In the USA, indeed, antagonistic positions between Democrats (politically liberal and generally pro-mask) and Republicans (politically conservative and generally anti-mask) on the mandatory use of masks soon turned their rejection into a symbol of political affiliation [4, 41]. This was in keeping with many political differences in wider attitudes towards the pandemic [3] and towards medical procedures orientated to public health, especially vaccinations [42]. Indeed, one study with US participants found that conservative political affiliation (along with younger age) was a major predictor of anti-mask attitudes [43].

Again, this fits with the predictions of moralization theory, which suggests that moralization of new social norms frequently occurs in the public health domain because violating the norm can be seen as harmful to others (Rozin, [30], focused on the example of the prohibition of cigarette smoking in public). This occurs by a process that Rozin called “moral piggybacking,” in which “new experiences or knowledge may cause a previously neutral activity or object to fall under an already functioning moral principle” (p. 219). However, if this new knowledge is not universally accepted, it can create intense moral disagreement. In the case of masks, political conservatives might be less accepting of the evidence for their positive effects on health—perhaps motivated by a desire to preserve their personal freedoms (see [44]) in the face of a seemingly alien cultural practice—and less trusting of their wearers, whom they might suspect of wanting to take their freedoms away.

Based on the reviewed literature, we suspected that political orientation might affect reactions that US and UK people have towards masked faces, leading to the following hypotheses:

- *H1:* There would be an interaction between mask-wearing and conservative political orientation, such that:
  - *H1A:* more conservative participants would see mask-wearers as less trustworthy;
  - *H1B:* more conservative participants would desire more social distance from mask-wearers;

We further predicted that both interactions would be significant when considering the effect for individual differences in social trust, social anxiety, and pathogen disgust sensitivity. We did not make any prediction for the effect of political orientation on sickness perception.

Additionally, we sought to clarify why masked faces were judged to be more trustworthy and less deserving of social distance in the study of Olivera-La Rosa et al. [23], despite the fact that mask-wearers were also perceived as more likely to be sick—in contrast to what the theory of the behavioral immune system would predict. One possibility is that the social norm of mask-wearing was strong enough to generate an affiliative reaction in viewers that overrode the aversion generated by a perception of sickness. Another is that the social norm generated a kind of social desirability bias that caused people to say they would want less social distance from the masked targets, but that this explicit judgement would not be reflected in their behavior. Hence, we investigated whether a more implicit, quasi-behavioral instrument (the online-VAAST approach/avoidance task; [45]) would show a pattern of results more similar to the explicit results for trustworthiness and desired social distance (quicker approach to masked faces than unmasked ones), or the explicit results for sickness perception (quicker avoidance of masked faces than unmasked ones). This led us to the following hypotheses:

- *H2:* Effects of condition would be seen in the online-VAAST task, such that:
  - *H2A:* there would be differences in the average time taken for approaching masked and unmasked faces.
  - *H2B:* there would be differences in the average time taken for avoiding masked and unmasked faces.
  - *H2C:* conservative voters would approach mask-wearers more slowly and avoid them more quickly, compared to liberals.

We made no prediction about the direction of either H2A or H2B, since as explained above, the results of the original study could support either a positive or negative difference, depending on the political profile of the participants and the relative strength of increased approach tendencies (due to greater trust) and increased avoidance tendencies (due to sickness cues). We tested H2C as well as the explicit hypotheses about political affiliation because of evidence that an individual’s degree of conservatism or liberalism does not necessarily affect their physiological reactions [46]; and that in threatening situations, liberals may react more like conservatives on some measures [47]. This leads to the question of whether differential affective reactions to social norms between political groupings appear at only an explicit level, or at both explicit and implicit levels (in line with theoretical and experimental evidence for the internalization and automatization of social norms; [6, 48]).

All main hypotheses and sub-hypotheses were pre-registered on the Open Science Foundation website.^1^ The data and code are also located at that link, along with an explanation of some minor deviations from the pre-registered analyses, which resulted from peer reviewers’ comments on the pre-registered design (see also Supplementary Information S0).

## Methods

### Participants

We recruited 1241 participants (711 women, 513 men, 19 preferred not to state binary gender) via the Prolific participant recruitment system [49]. Sample size was decided based on a power analysis for linear mixed models, based on the effect sizes found by Olivera-La Rosa and colleagues [23] and following the considerations presented by Judd et al. ([50], see Supplementary Information S1). The minimum effect size of interest was set at *d* = 0.1, corresponding to the smallest significant effect size reported by Olivera-La Rosa et al. [23]. Some 622 participants resided in the UK and 619 in the USA (see Table 1 for a summary of demographic data). Participants completed the experiment in an average of 13 min, receiving a fixed payment of GBP£2.10 or USD$2.87 in exchange for participating. Informed consent was obtained from all the participants. The study was conducted in accordance with American Psychological Association ethical principles and approved by the Ethics Committee of university name redacted for peer review.

**Table 1:** Summary of the sample’s demographic data, split by country

	UK (*n* = 622)	USA (*n* = 619)	Total (*N* = 1241)
	Value (col %)	Value (col %)	Value (col %)
Gender			
Female	410 (66.7%)	301 (48.6%)	711 (57.3%)
Male	205 (33.3%)	308 (49.7%)	513 (41.3%)
Other	7 (1.1%)	10 (1.6%)	17 (1.4%)
Age: mean [SD] in years	34.4 [12.7]	32.3 [12.1]	33.4 [12.5]
Education: highest level achieved			
Primary education	1 (0.1%)	34 (5.5%)	35 (2.8%)
Secondary education (<= 16 years)	40 (6.4%)	77 (12.4%)	117 (9.4%)
Professional qualification (> 16 years)	218 (35.0%)	125 (20.2%)	343 (27.6%)
Undergraduate degree	238 (38.3%)	245 (39.6%)	483 (.39%)
Postgraduate degree	105 (16.9%)	122 (19.7%)	227 (18.3%)
Doctorate	20 (3.2%)	16 (2.6%)	36 (2.9%)
Politics			
0–10 liberal–conservative: mean [SD]	4.15 [1.93]	3.88 [2.34]	4.02 [2.15]
Voting intention			
Labour/Democrat candidate	335 (53.8%)	425 (68.6%)	760 (61.2%)
Conservative/Republican candidate	122 (19.6%)	134 (21.6%)	256 (20.6%)
Other candidate	165 (26.5%)	60 (9.7%)	225 (18.1%)

### Materials

The experiment was performed online using *PsyToolkit* [51]. First, participants indicated their age, sex, highest educational qualification, country of residence, and voting preference in national elections (Supplementary Information S6). They then completed one block each of (a) explicit and (b) implicit measures, as well as (c) answering a set of individual differences questionnaires. Blocks (a) and (c) were adapted from the materials used by Olivera-La Rosa et al. [23], with the main changes being the translation from Spanish to English, the addition of female as well as male faces to block (a), and the addition of questions on political orientation and voting intention to block (c). For the explicit measures block (a), we used a randomized passive control group design, with one factor that had two possible levels (masked or unmasked faces) randomly allocated to two equal-sized groups. A between-groups design was used in order to reduce the possible demand effects of being asked to evaluate both masked and unmasked faces. For the implicit measures block (b), we used a within-subjects design that had one factor with two levels of instructions (one trial where participants were instructed to approach masked faces and avoid unmasked ones, and another trial vice versa). For the individual differences block (c), all participants received exactly the same questions.

**Fig. 1:**
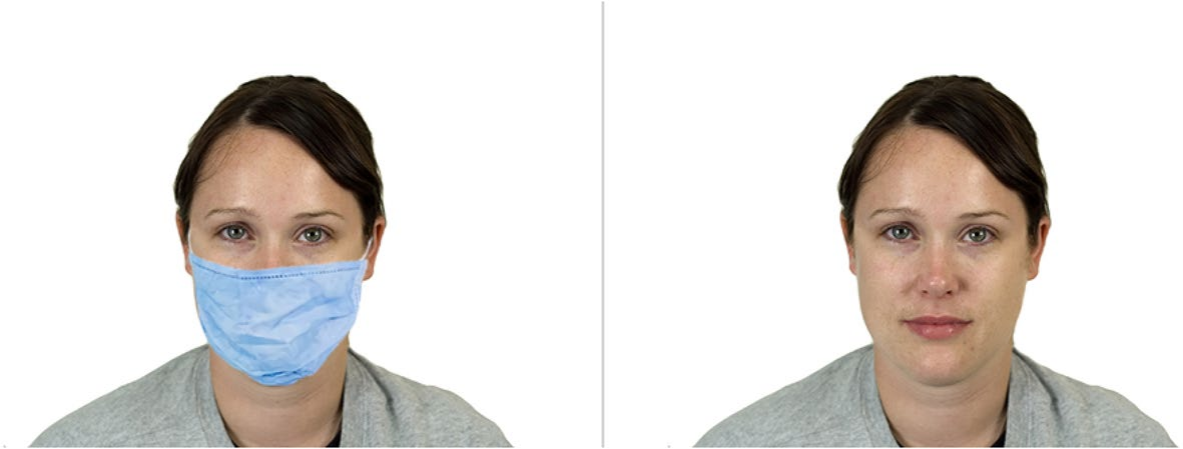
Example of Target Face*. Note.* The photographs show one of the ten faces from the Chicago Face Database used in the experimental—masked—condition (left) and control condition (right), for the explicit methods in block (a)

*Explicit measures block (a).* While viewing each of ten target faces, participants were asked to respond to three questions relating to social perception (Supplementary Information S7). As targets we selected five male and five female faces from the Chicago Face Database [52] with the inclusion criteria of neutral emotional expressions and average ratings on the aesthetic dimension. Male faces were four Latino faces and one Black face (mean age 25.06 years; the number of Latino faces was due to adapting the design used by Olivera-La Rosa et al., [23], in Latin America and Spain). Female faces consisted of three White faces, one Latino face and one Black face (mean age 24.58 years). For the experimental condition, we added a blue surgical-style mask to each face using Adobe Photoshop. The same faces without masks were displayed in the control condition. Therefore, in this block participants saw only one version (masked or not) of each face, always either masked or unmasked depending on the condition (see Fig. 1).

For each face, participants first completed the Social Distance Scale [53]. In this measure, participants indicated the “closest” level of interaction with which they would feel comfortable with the evaluated person, on a 7-point scale (e.g., “I would feel comfortable if this person were a close friend”): a higher score indicated that more social distance was desired (Supplementary Information S7). They then rated the target’s trustworthiness on a 7-point scale (“Based on your initial reaction, how trustworthy does this person seem to you?”). Finally, participants evaluated (with “Yes” or “No”) the perceived sickness or healthiness of each face (“Based on your initial reaction, do you think that this person is sick or healthy?”).

**Fig. 2:**
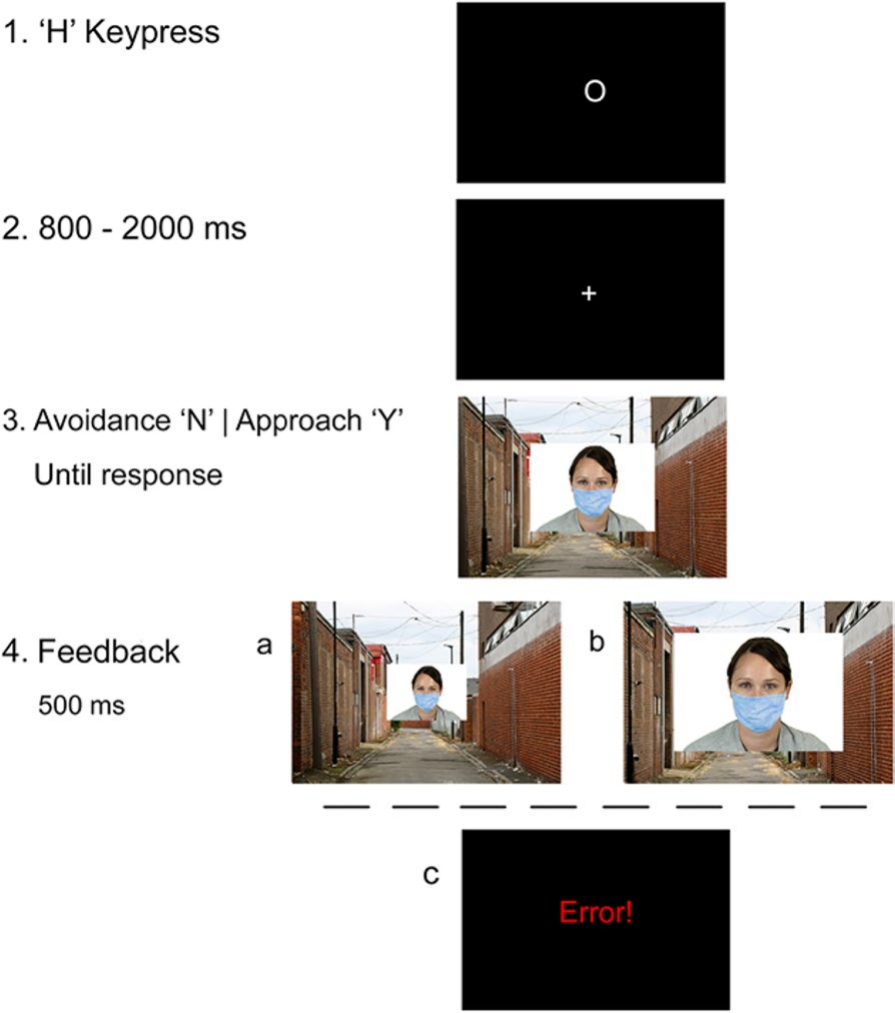
Trial Sequence in Block (b), the Online-VAAST*. Note.* In this trial the target is wearing a mask. In one sub-block, the correct response is an avoidance action (< N > keypress), whereas in the other sub-block, the correct response is an approach action to the same face (< Y > keypress). Participants received feedback for each trial. With correct avoidance responses, the background and the target face appeared to recede (4a), whereas with correct approach responses, the background and the face appeared to get closer (4b). With incorrect responses, participants received an “Error!” message as feedback (4c)

*Implicit measures block (b).* We adapted the online Visual Approach/Avoidance by the Self Task (online-VAAST [45];). In this task, participants responded to targets as quickly as possible with a quasi-behavioral measure. They were instructed to perform either an approach or an avoidance action (by pressing < Y > or < N > on their computer keyboard: < Y > made the target appear to get closer, whereas < N > made it appear to recede; Fig. 2). The task had two sub-blocks. In one, participants were instructed to approach faces wearing a mask and to avoid faces without a mask. In the other, they were instructed to approach faces without a mask and to avoid faces wearing a mask. Each sub-block presented the same ten faces with and without a mask, against the same background of a side alley, in random order, until participants had responded to all of them (20 trials in total).

*Individual differences block (c).* We gave participants questionnaires on social trust, pathogen disgust sensitivity, and social anxiety. To measure social trust, we deployed the widely used Standard International single-item scale [54]. Participants were asked to indicate with a score of 0–10 (ranging from 0, “You can’t be too careful,” to 10, “Most people can be trusted”) their answer to the following statement: “Generally speaking, would you say that most people can be trusted, or that you can’t be too careful in dealing with people?” Following the suggestion of Aarøe et al. [18], we combined this score with the six-item General Trust Scale [55]. For the latter measure, participants were asked to indicate how much they agreed or disagreed with six statements (e.g., “Most people are basically honest”; Supplementary Information S8) using a five-point scale from “Strongly disagree” to “Strongly agree,” with higher values indicating greater trust.

To measure disgust sensitivity, we used the Pathogen Disgust subscale of the Three-Domain Disgust Scale [56]. This subscale comprised 7 items, for which participants indicated on 7-point scales how disgusting they would find a series of situations relating to infectious agents (e.g., “Accidentally touching a person’s bloody cut”; Supplementary Information S9). This subscale has been used in previous research on pathogen-associated motivations [21, 57]. As a measure of social anxiety, we applied the Avoidance subscale of the Liebowitz Social Anxiety scale [58]. In this questionnaire, participants were asked to rate their tendency to avoid each of 24 social situations (e.g., “Drinking with others”; Supplementary Information S10) on a 4-point scale.

### Procedure

Data was collected in October 2020. After giving informed consent and answering the demographic questions, which always came first, participants received the three main blocks of the experiment in two possible orders: (i) either explicit judgements of masked or unmasked faces, or the implicit approach/avoidance task; (ii) the individual differences survey questions on social anxiety, generalized social trust, and pathogen disgust sensitivity; and (iii) either the implicit approach/avoidance task or explicit judgements of faces (whichever task they did not receive in (i)). We randomized the order of the faces for the explicit questions, along with the order of presentation of the three sets of individual differences questions (on pathogen disgust sensitivity, social anxiety, and generalized social trust), and the two sub-block combinationliebs of approach/avoid and masked/unmasked faces in the implicit task.

### Data exclusion

Individuals (*n* = 8) who failed both of two attention check questions (see Supplementary Information S3) were excluded completely from the analysis. Individuals who had a high Cook’s distance (using an algorithm presented by [59] which yielded a cut-off point of 0.00326) on any of the dependent measures were excluded for that measure alone (*n* = 44 for trustworthiness; *n* = 33 for sickness perception; *n* = 43 for social distance). For the online-VAAST implicit task, participants were filtered by error rate, such that those with less than 60% success in following the instructions were completely excluded on the reaction time measures (*n* = 31), as were 3594 incorrect trials from participants whose correct trials were still included. As in other studies of face perception [60], 55 trials with a reaction time of less than 200 ms were also excluded from the implicit measures analysis. Furthermore, following Aubé and colleagues’ [45] application of the online-VAAST, 929 trials with reaction times above 2500 ms were also excluded from this analysis. Participants excluded by the implicit measures criteria detailed in this paragraph were still included in the tests of explicit measures (*N* = 1189 for trustworthiness; *N* = 1200 for sickness perception; *N* = 1190 for social distance; *N* = 1202 for reaction times).

## Results

Participants’ explicit judgments (*H1*) were analyzed using linear mixed-effects models, which account for both between-subjects and within-subjects effects of independent variables ([61]; see Supplementary Information S11 for more details). We fitted three models for each question about the target faces (trustworthiness, perceived sickness, and social distance). The first set of models (Table 2) examined the interaction of *Condition* (Masked or Unmasked target) with *Political Orientation* scores, which were centered on their grand mean and included as a continuous predictor. The second set of models included the main effects of *Pathogen Disgust Sensitivity*, *Social Anxiety*, and *Generalized Social Trust*, and the interaction of *Condition* with these scales. Internal consistency was excellent for disgust sensitivity (Cronbach's *α* = 0.85, *M* = 4.33, *SD* = 1.16), social anxiety (*α* = 0.91, *M* = 1.38, *SD* = 0.57), and social trust (*α* = 0.89, *M* = 0.64, *SD* = 0.16). Results of the second set of models are described in Table 2. (In Supplementary Information S20, we present a third set of models with the additional inclusion of demographic variables, for which we had no hypotheses, and which did not affect the overall pattern of results described in this section.) *Participant* and *Stimulus* (i.e., the individual faces evaluated) were included as random effects in all models. For all significance tests, we pre-registered an alpha value of 0.01, obtained using the Satterthwaite method of alpha correction for repeated tests [62]. All tests between conditions were two-tailed.

**Table 2:** Main effects and interactions of the models containing the hypothesized predictors of the explicit measures (Model 1) and hypothesized predictors alongside covariates marking individual differences between participants (Model 2)

**Model 1:**	Trustworthiness			Sickness perception			Social distance		
**Hypothesized effects**	*β*	*t*	*p*	*β*	*Z*	*p*	*β*	*t*	*p*
Condition	.032	4.94	< .001	-.16	-1.18	.24	.0146	2.22	.033
Political orientation (PO)	-.0068	-3.90	< .001	.061	2.32	.020	.015	6.44	< .001
Condition * PO	-.0045	-2.56	.010	.066	2.48	.013	.0019	.83	.41
**Model 2: Full effects**									
Condition	.060	9.02	< .001	-.34	-3.35	< .001	.027	2.10	.043
Political Orientation (PO)	-.011	-4.73	< .001	.097	2.90	.0037	.017	4.36	< .001
Disgust Sensitivity (DS)	-.0010	-1.66	.096	-.015	-1.82	.068	.00003	.035	.97
Social Anxiety (SA)	.0002	.56	.57	.016	3.18	.0014	.0006	1.08	.28
Social Trust (ST)	.26	8	< .001	-.76	-1.66	.097	-.18	-3.72	< .001
Condition * PO	.0091	2.81	.0051	-.094	-2.13	.033	.0049	-1.02	.31
Condition * DS	.0008	.97	.33	.031	2.75	.006	.0034	2.85	.0044
Condition * SA	-.0003	-.63	.53	-.013	-1.94	.052	.0004	-.56	.58
Condition * ST	.021	.49	.62	-.28	-.48	.63	.065	1.06	.29

### Trustworthiness

**Fig. 3:**
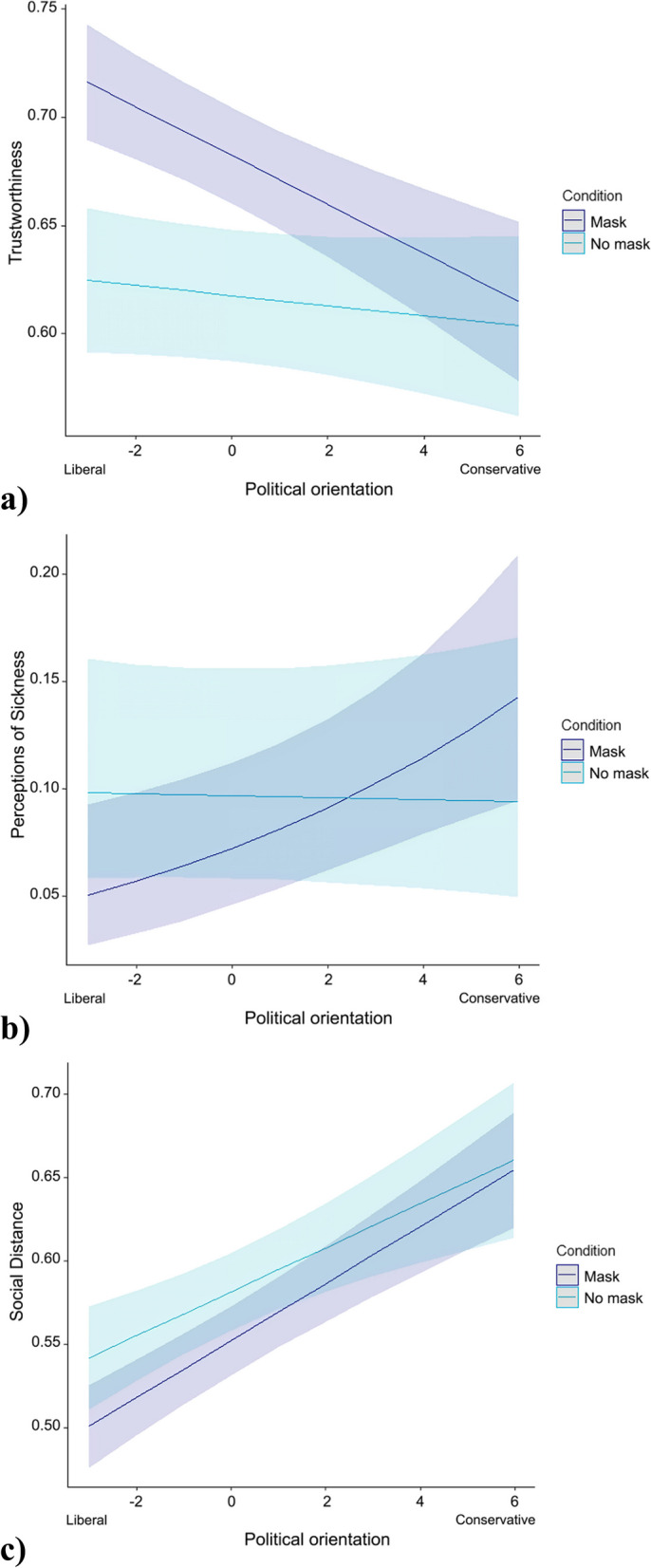
Model-Predicted Relationships between **a**) Trustworthiness, **b**) Perceived Sickness and **c**) Preferred Social Distance of Masked and Unmasked Targets, Depending on Political Orientation. *Note*. Shaded areas represent 95% CIs

A *t-*test of simple effects showed that participants were moderately more likely, *t* (1239) = 9.01, *p* < 0.001, *d* = 0.50, to trust targets with a mask (*M* = 4.81, *SD* = 0.95, 95% CI [4.74, 4.89], *n* = 592) than targets without a mask (*M* = 4.34, *SD* = 0.92, 95% CI [4.27, 4.41], *n* = 649). As set out in Table 2, in the linear model analysis the main effect of political orientation indicated that conservatives had less trust in the target faces. The interaction with condition revealed that this effect was mainly explained by their lower trust for targets with a mask. A simple slope analysis showed that participants’ political orientation was not significantly associated with trustworthiness in the control condition (Slope = -0.01, *SE* = 0.02, *t* = -0.97, *p* = 0.33), whereas in the experimental (masked) condition more liberal participants tended to judge the faces as more trustworthy (Slope = -0.02, *SE* = 0.01, *t* = -4.46, *p* = 0.001; see Fig. 3a). All these effects remained significant in the full model controlling for individual differences (see Table 2, Model 2), and a main effect of generalized social trust was also found.

### Perceptions of sickness

Participants did not perceive targets with a mask (*M* = 0.152, *SD* = 0.187, 95% CI [0.138, 0.167], *n* = 592) as significantly more likely to be sick than targets without a mask (*M* = 0.178, *SD* = 0.184, 95% CI [0.164, 0.192], *n* = 649). Indeed, simple effects analysis indicated that any effect was more likely in the other direction, with unmasked people perhaps appearing slightly more likely to be sick, *t* (1239) = 2.44, *p* = 0.015, *d* = 0.14, but this effect was small and did not quite reach significance according to the Satterthwaite-corrected alpha level of 0.01; nor was it significant in the linear model analysis including political orientation (see Table 2). On the subject of political orientation, conservatives perceived targets in general as more likely to be sick than did liberals. As with trustworthiness, the interaction with condition revealed that this effect was largely explained by their perceptions of masked targets, with a steeper slope for participants in the treatment condition (Treatment Slope = 0.13, *SE* = 0.04, *z* = 3.04, *p* < 0.001 vs Control Slope = -0.01, *SE* = 0.03, *z* = -0.16, *p* = 0.88; see Fig. 3b). In the full model with individual differences included, these effects were preserved, and there were also significant main effects of condition and social anxiety, as well as a significant interaction between disgust sensitivity and condition (see Table 2, Model 2).

### Social distance

The desired social distance for targets with a mask (*M* = 3.83, *SD* = 1.29, 95% CI [3.72, 3.93], *n* = 592), was significantly lower than that for unmasked targets (*M* = 4.03, *SD* = 1.21, 95% CI [3.94, 4.12], *n* = 649), in the simple effects analysis, *t* (1239) = 2.88, *p* = 0.004, *d* = 0.16. However, the effect was small, and in the linear model including political interaction, it was not significant according to the corrected alpha level of 0.01 (see Table 2). The significant main effect of political orientation and lack of a significant interaction with condition indicated that conservatives reported more desired social distance from targets, regardless of whether or not they were wearing a mask (in other words, there was no significant interaction between experimental condition and political orientation). Both the main effect and the absence of an interaction were preserved in the second model including individual differences (shown in Table 2, Model 2), where an unexpected main effect of generalized social trust on desired social distance, as well as an interaction between condition and pathogen disgust sensitivity, were also detected. Social anxiety had no significant main effects on any of the dependent measures, or significant interactions with any other independent variable in the models.

### Approach/avoidance reaction times

**Fig. 4:**
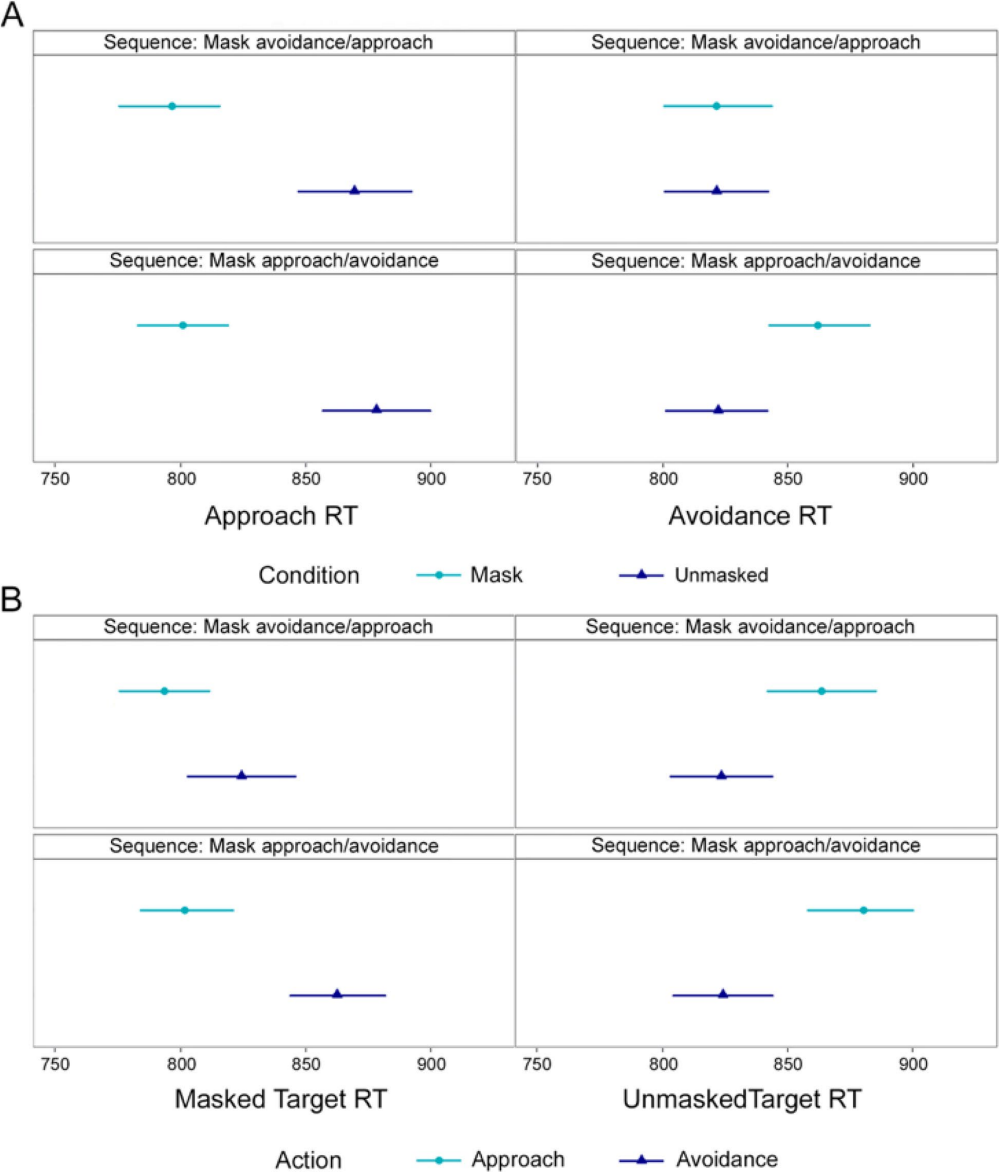
Mean Reaction Times (RTs) in the Online-VAAST*. Note.* **A** Left side shows approach RTs to masked and unmasked targets in each sequence. Right side shows avoidance RTs to masked and unmasked targets in each sequence. **B** Left side shows masked targets RTs with approach and avoidance actions in each sequence. Right side shows unmasked targets RTs with approach and avoidance actions in each sequence. Error bars represent 95% CI around the means

For the online-VAAST implicit measures (*H2*) we fitted four random-effects models, summarized in Fig. 4. Two models focused on the comparison between RTs (reaction times) to targets with and without a mask when performing approach and avoidance actions (Table 3). They included *Target* (Masked or Unmasked), and *Sequence* (Approach First or Avoidance First) as categorical fixed effects. The other two models focused on the comparison between approach and avoidance RTs to targets with and without a mask (Table 4). These included *Action* (Approach or Avoidance), and *Sequence* (Approach First or Avoidance First). All models also included *Political Orientation* as a continuous predictor, and participant and stimulus as random effects.

**Table 3:** Model results for approach/avoidance actions between masked and unmasked targets

Models	Approach action			Avoidance action		
**Fixed effects**	*β*	*t*	*p*	*β*	*t*	*p*
Target: Masked vs Unmasked	70.57	7.47	< .001	2.53	.28	.77
Sequence	16.66	1.19	.23	.29	.022	.98
Political Orientation	9.15	2.81	.005	12.63	4.08	< .001
Target * Sequence	-6.39	-.58	.56	36.85	3.08	.002
Target * Political Orientation	.57	.22	.82	-7.22	-2.59	.009

In the Approach action model (see Table 3), participants reacted faster to masked (*M* = 797 ms, *SD* = 247.2 ms, 95% CI [783 ms, 811 ms]) than unmasked targets (*M* = 871 ms, *SD* = 259 ms, 95% CI [854 ms, 888 ms]). More liberal participants approached both types of target faster than did more conservative participants. In the avoidance action model, there was no overall effect of target, though there was a significant interaction between target and sequence. More liberal participants avoided both types of target faster than did more conservative participants. However, the interaction with target revealed that this effect was larger for unmasked targets than for masked ones.

In the Masked targets model (Table 4), participants reacted faster with approach (*M* = 797 ms, *SD* = 283 ms, 95% CI [783 ms, 811 ms]) than avoidance actions (*M* = 843 ms, *SD* = 284 ms, 95% CI [828 ms, 857 ms]). In the Unmasked targets model, participants were faster at avoidance (*M* = 822 ms, *SD* = 282 ms, 95% CI [809 ms, 836 ms]) than approach actions (*M* = 871, *SD* = 308.6, 95% CI [854 ms, 887 ms]). Both approach and avoidance RTs to unmasked targets were faster for more liberal participants than for conservatives (Approach Slope = 9.51, *SE* = 3.25, *t* = 2.81, *p* = 0.01; Avoidance Slope = 12.71, *SE* = 3.10, *t* = 4.10, *p* < 0.001).

**Table 4:** Significant effects and interactions of the models predicting the reaction times of different actions for masked and unmasked targets

Models	Masked target			Unmasked target		
**Fixed effects**	*β*	*t*	*p*	*β*	*t*	*p*
Action: Approach vs Avoidance	-32.97	-3.52	< .001	39.93	4.39	< .001
Sequence	37.52	2.68	0.007	0.22	0.02	0.98
Political orientation	5.02	1.33	0.19	12.71	4.10	< .001
Action * Sequence	-26.19	-2.30	0.02	16.84	1.45	0.15
Action * Political orientation	4.49	1.34	0.19	-3.56	-1.31	0.19

## Discussion

We tested whether reactions to masked faces co-varied with political orientation in the UK and US populations, in a pre-registered online experiment using both explicit and implicit methods. This study was important since as far as we know, it is the only published study with data collected in the pandemic on the effects of political orientation on reactions to the visual presentation of masked faces. Indeed, we found that country of origin was not a significant predictor in our model, but political orientation was, with the overall positive effect of mask-wearing on trustworthiness being driven by liberal participants’ reactions. Another important feature of our design was the integration of explicit and implicit methods, with the pattern of responses being quite similar between the two, for both liberal and conservative participants. Again, this is the only study we know of that examined implicit attitudes to masked faces during the COVID-19 pandemic, which is important for understanding whether people were internalizing political messages about mask-wearing being a socially desirable practice.

The positive effects of masks on trustworthiness found by Olivera-La Rosa et al. [23] replicated in our sample as a medium-sized effect, demonstrating some international consistency in attitudes towards masked faces. However, the effect on preferred social distance was small and was not robustly significant once political orientation was taken into account. The negative effect of masks on perceptions of healthiness found by Olivera-Rosa and colleagues disappeared or even reversed, perhaps reflecting the newfound ubiquity of masks in that they are now seen as an everyday object that helps protect against contagion, rather than potential indicators of disease (cf. [39], who argued that masks had a less negative effect on facial attractiveness in a pandemic context than before the pandemic). There were also individual differences in the acceptance of masks: in line with *H1A*, liberals showed more trust in masked faces (relative to unmasked ones) than did conservatives, and were less likely than conservatives to view mask-wearers as sick. However, these differences did not translate into a greater desired social distance towards mask-wearers on the part of conservatives, leading to the rejection of *H1B*. The political differences in evaluations that we did find may reflect both the continued controversy over the use of masks in public, and the potential for political orientation to affect one’s judgments of strangers, depending on the symbols of affiliation that they display [63].

The overall positive explicit judgments of mask-wearers were mirrored in the reaction time scores, which showed that people were faster to approach masked faces than unmasked faces (supporting *H2A*), and faster to approach than avoid masked faces (but faster to avoid than approach unmasked faces). However, there was no significant difference between masked and unmasked faces in terms of avoidance reaction time, leading to the rejection of *H2B*. The coherence of these implicit results using the online-VAAST approach/avoid task [45] with those obtained using explicit judgments support the external validity of this measure, suggesting that in some contexts it may be a useful alternative to better-known implicit measures such as the IAT (Implicit Association Test; cf. Rougier et al., [64]), which has been much criticized of late [65–67]. Furthermore, in line with *H2C*, political conservatives were relatively quicker to avoid masked faces than liberals were, mirroring the differences found in their trustworthiness and sickness judgments, and implying that political opinions might have the potential to impact behavior in unconscious ways. This result contrasts with a recent study that found similarities in unconscious reactions to threatening phenomena between liberals and conservatives [46], and supports the idea of a general congruence between implicit and explicit attitudes [48].

Considering that a key objective was to assess the effects of political orientation on responses to masked faces, a limitation of the current study was an over-representation of liberal (c. 60%) compared to conservative (c. 20%) voters. This was likely due in part to over-representation of women, young people and highly-educated people relative to the UK/US populations in general, since all these groups have more liberal voting tendencies than the national averages [68]. Other limitations concerned the dependent measures that we used. As already pointed out by Olivera-La Rosa et al. [23], the self-reported measure of desired social distance was rather indirect and of unclear validity (its deployment as an ordinal measure relies on the assumption that people conceive of relationships such as family, friends, neighbors and colleagues in an objectively ranked way, whereas phenomenologically these may be experienced more like a nominal variable, and different levels’ closeness rankings may vary between individuals). This may have contributed to the trustworthiness measure showing stronger effects, even though it too was based on self-report.

The current research could be extended by including additional control conditions (e.g., using images that only showed the area around the eyes) that allow us to clarify whether the higher trustworthiness observed for masked faces was due to a specific effect of masks, as opposed to hiding part of the face. Previous research suggests that, when they encounter novel faces, humans typically combine signals of social category membership or norm affiliation (e.g., from clothing) with implicit judgements of facial expressions, in complex and not easily predictable ways [10, 69, 70]. Along these lines, an interesting topic for further research to pursue would be the role of holistic visual processing: we know that this is impaired in the perception of masked faces [34, 35], but also that evaluations of trustworthiness depend on holistic processing [71], so it seems counterintuitive that masking a face would increase its trustworthiness. This suggests that culturally charged symbols such as masks may sometimes be included in the holistic visual processing involved in social judgements like those relating to trust, a hypothesis that might be tested using the composite-face paradigm employed by Todorov and colleagues.

Behavioral measures of reactions to masked faces, such as an economic trust game, or assessment of how much people believe statements spoken by each face, might show more ecological validity, as well as being interesting outcomes for further investigation in their own right. Indeed, a recent study by Malik et al. [72] found that fewer members of their US sample trusted information that was given to them by someone wearing a mask. Although superficially contradicting our results, one key difference was that these authors used video stimuli (with audio). Trust towards a masked *speaker* might be particularly reduced, since the hiding of facial expressions and muffling of tone could impair processing of meta-conversational information by the audience. In a similar vein, Grundmann and colleagues [32] found that evaluations of trustworthiness and desired social distance of mask-wearers were affected by the emotional expressions of control faces. Perhaps the neutral expressions worn by our controls were perceived as somewhat “cold,” contributing to their lack of trustworthiness. Supporting this interpretation, Marini et al. [73] found that when evaluating static photos of faces wearing opaque (but not transparent) masks, participants reported lower trust in masked faces showing the emotions of happiness, sadness and fear (compared to unmasked control faces showing the same emotions), but that the same pattern did not apply to neutral faces. Future studies of the impact of masks on face perception should take into account this apparent sensitivity of reactions to the emotional expressions of the control faces that are used.

The precise nature of the dependent measures that are used is likely to be another source of variability of results in this kind of study. With respect to political orientation, we did find some coherence between explicit and implicit measures. This is in line with a recent theoretical model of the dynamic, cooperative organization of controlled and automatic processes in the human mind, which helps to produce habitual behavior in accordance with social norms [74]. Further hypothesis-driven exploration of our open data, as well as follow-up studies, could be performed to investigate how both sets of measures intercorrelated and how they varied between different subgroups of participants. That this congruence was seen more clearly in approach than avoidance reaction times is also interesting. We did not anticipate this finding, but one explanation, in the context of the online-VAAST task, might be that “approaching” a face is more ecologically valid than “avoiding” it, since in the framing of this task “approaching” a face would correspond to walking towards them, whereas “avoiding” it would correspond to making them recede by walking backwards, when a more natural avoidance action might be to turn away and follow a different path.

Generalizing from the current research on mask use, since moralization is thought to be a typical result of the introduction of new social norms relating to morally charged areas such as health, it is likely that there may also have been moralization of other novel norms in the context of COVID-19, such as attitudes to vaccines [75–77]. It would thus make sense to investigate whether estimations of someone’s likelihood to have been vaccinated (e.g., based on their political profile) affect explicit judgments of their trustworthiness, healthiness, or social desirability, as well as implicit reactions [42].

In conclusion, our results support the idea that new social norms such as mask-wearing can quickly come to affect people’s evaluations of others, even at an automatic level [6], and form fault lines for moralized political differences [78]. As Prosser and colleagues [79] pointed out, in the face of political or identity-based differences in the acceptance of novel health-related norms, it is important to avoid exacerbating social divisions by labeling those who do not immediately accept these norms as “immoral”—a practice that is all too common in both traditional and social media. Rather, to promote a more generally accepted moralization of life-saving practices, it is vital that people are given the chance to negotiate and buy into the novel norms themselves, perhaps by showing them how they are linked to moral values that they find particularly important [80]. Research into how signals of norm affiliation relate to judgements of trustworthiness and related qualities could be a key input to this process—not only in the context of the COVID-19 pandemic, but also applied to equally polarized contemporary debates, such as those surrounding climate change.

### Supplementary Information

**Additional file 1: S0.** Deviations from pre-registration. **S1.** Sampling plan and power analysis. **S2.** Stopping Rule. **S3.** Data Exclusion. **S4.** Blinding. **S5.** Statement of informed consent. **S6.** Demographic questions. **S7.** Experimental materials. **S8.** Standard International and Generalized Trust scales. **S9.** Three-Domain Disgust Scale (Pathogen Disgust subscale). **S10.** Liebowitz Social Anxiety scale (Avoidance subscale). **S11.** Analytical strategy. **S12.** Complete models predicting trustworthiness judgments. **S13.** Complete models predicting sickness judgments. **S14.** Models predicting social distance judgments. **S15.** Model predicting implicit Approach action to targets in the O-VAAST. **S16.** Model predicting implicit Avoidance action to targets in the O-VAAST. **S17.** Model predicting implicit actions to targets with mask in the O-VAAST. **S18.** Model predicting implicit actions to targets without mask in the O-VAAST. **S19.** Correlations between results from the explicit judgements and the O-VAAST. **S20.** Demographic effects on the explicit measures. **S21.** Political orientation and sickness perceptions. **S22.** Target gender and sequence effects on the implicit measures. **S23.** Models predicting the influence of political orientation and individual differences on explicit judgements. **S24.** Comparison analyses to assess the influence of political orientation and voting intention in the explicit judgements. **S25.** Comparison analyses to assess the influence of political orientation and voting intention in the implicit O-VAAST. **S26.** Correlations between results from the explicit judgements and the O-VAAST. **S27.** R code and output used to calculate observed power and to derive inputs for the standardized calculation of VPCs.

## Data Availability

The datasets generated and/or analysed during the current study are available in the Open Science Framework repository, https://osf.io/3bpdw/?view_only=fc1bfbfab1df48b6ac247d5d5e581542

## Footnotes

1 https://osf.io/3bpdw/?view_only=fc1bfbfab1df48b6ac247d5d5e581542
