# Supplementary material for: In masks we trust: explicit and implicit reactions to masked faces vary by political orientation
Source: BMC Psychol. 2024 Feb 12;12:68. doi: 10.1186/s40359-024-01556-5 (PMC10863087; doi:10.1186/s40359-024-01556-5)
Supplement: Supplementary file 1 — Additional file 1: S0. Deviations from pre-registration. S1. Sampling plan and power analysis. S2. Stopping Rule. S3. Data Exclusion. S4. Blinding. S5. Statement of informed consent. S6. Demographic questions. S7. Experimental materials. S8. Standard International and Generalized Trust scales. S9. Three-Domain Disgust Scale (Pathogen Disgust subscale). S10. Liebowitz Social Anxiety scale (Avoidance subscale). S11. Analytical strategy. S12. Complete models predicting trustworthiness judgments. S13. Complete models predicting sickness judgments. S14. Models predicting social distance judgments. S15. Model predicting implicit Approach action to targets in the O-VAAST. S16. Model predicting implicit Avoidance action to targets in the O-VAAST. S17. Model predicting implicit actions to targets with mask in the O-VAAST. S18. Model predicting implicit actions to targets without mask in the O-VAAST. S19. Correlations between results from the explicit judgements and the O-VAAST. S20. Demographic effects on the explicit measures. S21. Political orientation and sickness perceptions. S22. Target gender and sequence effects on the implicit measures. S23. Models predicting the influence of political orientation and individual differences on explicit judgements. S24. Comparison analyses to assess the influence of political orientation and voting intention in the explicit judgements. S25. Comparison analyses to assess the influence of political orientation and voting intention in the implicit O-VAAST. S26. Correlations between results from the explicit judgements and the O-VAAST. S27. R code and output used to calculate observed power and to derive inputs for the standardized calculation of VPCs. [file 40359_2024_1556_MOESM1_ESM.docx]

**In Masks we Trust:**

**Explicit and Implicit Reactions to Masked Faces Vary by Political Orientation**

**Supplementary Information**

## Author Contributions

*[Redacted for peer review.]*

## Methods

### S0. Deviations from pre-registration

All main hypotheses and sub-hypotheses were pre-registered on the Open Science Foundation website^^[[1]](#footnote-1)^^.In order to make the published manuscript clearer, we removed the hypothesis described as H1ABC in the preregistration, because it simply described the replication of the results in the study of Olivera-La Rosa et al. (2020), which is implied in the re-use and extension of their methods in the present study. This led us to renumber H3AB as H1AB, and H3C as H2C in the main manuscript. Secondly, we had pre-registered hypotheses H3ABC in terms of the effects of voting intention, but following the suggestion of a reviewer of the pre-registered methods we collected data on political orientation as well as voting intention, and following the suggestion of a reviewer of the current manuscript we only report data on political orientation in the main manuscript (data on voting intention, which produced broadly similar effects but was a less sensitive measure, is included later in the Supplementary Information).

### S1. Sampling plan and power analysis

The sampling plan called for at least 1200 participants to be recruited. Inclusion criteria were that participants must be over 18 years old, have at least a 75% approval rate on the platform, be fluent in English, and currently reside in the UK or USA (600 from each country). To justify the sample size, we ran a power analysis for linear mixed models based on the estimates found by Olivera-La Rosa and colleagues (2020), and following the considerations and calculations presented by Judd, Westfall, and Kenny (2017). Linear mixed models are a powerful tool for dealing with dependencies in data and are increasingly used in psychological research (Baayen, Davidson & Bates, 2008; Judd et al., 2017; Meteyard & Davies, 2020; Snijders & Bosker, 2012). There are, however, disagreements on their reporting and interpretation, and power analyses for these models are notoriously complex and require a clear specification of the model (Meteyard & Davies, 2020). We therefore conducted two complementary analyses to make a decision on the sample size needed to achieve 95% statistical power. First, we manually derived effect sizes and Variance Partitioning Coefficients (VPC) from the models fitted by Olivera-La Rosa et al. (2020), taking the model for *trustworthiness* (since this showed the smallest effect of the three dependent variables) as the input to the power calculations outlined by Judd et al. (2017). Second, we calculated the observed power for the same study, using a simulation method implemented in the SIMR R library (Green & MacLeod, 2016).

The original study examined the effect of condition (masked versus non-masked faces) on perceived trustworthiness, social desirability, and sickness (Olivera-La Rosa et al., 2020). As such, participants and stimuli are treated as random factors, since they are both independent sources of variation for each data point. Although this design is fully crossed, with participants judging the same faces in both conditions, VPCs for random slopes are not included in the power analysis calculation. This is because these random slopes are very small (< 0.01) and do not contribute to the overall model fit. However, the experimental design does include contribution of random effects of participants and faces to the mean difference between conditions. Leaving aside these random slopes, then, there are three VPCs to be estimated:

$${VPC}_{Int.Participant}\frac{0.53}{0.53+0.019+1.13}=0.311$$

$${VPC}_{Int.Face}\frac{0.03}{0.53+0.019+1.13}=0.019$$

$${VPC}_{Residual}\frac{1.13}{0.53+0.019+1.13}=0.668$$

The effect size *d* was also derived following the method of Judd et al. (2017):

$$d= \frac{0.15}{\sqrt{0.53+0.019+1.13}}=0.115$$

These inputs were then used to calculate power with standardized and unstandardized coefficients. The first analysis suggests a sample size of 495 participants to achieve 95% power with 5 faces and the VPC’s above. The second analysis suggest at least 492 participants to achieve the same power with the same number of stimuli, using the raw variance estimates from the fitted model. We aim to collect data from at least 600 participants (after applying the exclusion criteria specified below) in each country to make sure we can detect an effect size of at least *d* = 0.1 even when the data is split by country.

We also ran a simulation based on the actual data for trustworthiness judgments in the study under replication (see Supplementary Information, Appendix G for the R code and output). The resulting observed power was 100% under 100 iterations of the simulation, indicating that the target study (Olivera-La Rosa et al., 2020) was well powered with an *N* of 1078 (slightly less than the 1200 proposed for the current study), assuming a true effect size *d* of 0.11. For the novel hypotheses *H2A* and *H2B*, concerning the implicit measures, potential effect size is unknown. However, methods used to test these two hypotheses will be within-subjects, so the power calculated here for the between-groups testing of *H1A* should be sufficient to detect any meaningful effect for those hypotheses too, given that within-subject designs are more efficient than the corresponding between-subject designs. Potential effect sizes for the novel hypotheses *H3A*, *H3B* and *H3C*, concerning political orientation, are also unknown, but may be relatively large. However, given that the predicted effect is an interaction, which requires more power (Giner-Sorolla, 2018), and will be tested between sub-groups of the experimental and control groups, it is possible that the testing of these hypotheses will be underpowered. Nevertheless, we will perform post hoc power analyses for the interaction to see whether the model had enough power to detect the interaction, whether or not we find a significant effect, under different assumptions of true effect size (Aguinis, Gottfredson, & Culpepper, 2013). In a sense, analyses of these hypotheses will thus be partly exploratory, and any notional effect sizes generated should be interpreted with caution; nevertheless, we wanted to state our predictions explicitly beforehand, since they have a clear directionality.

### S2. Stopping Rule

We stopped collecting data as soon as possible after receiving 600 participants in each country. We did not look at patterns within the data (e.g., *p* values) before deciding when to stop. We did however look at the data after running about 100 participants, to check that the survey worked well with Prolific and that the exclusion criteria made sense.

### S3. Data Exclusion

We included two attention check questions, one in the middle and one at the end of the study presentation. These were questions where the participant was required to give an incorrect answer based on instructions in the question context:

1. This question is simple. You need to give an alternative response to what you know about the sky. Please, make sure to select “Orange” as the answer to the next question so that we know that you understand the task properly.

What color is the sky? Blue | Orange

1. In this question, you have to choose which activity you prefer from a list. Regardless of the other options, please, make sure to select handball as your answer so that we know that you understand the task properly.
   Which activity do you prefer? Rugby | Baseball | Handball | Basketball | Cycling

Individuals who failed both questions were excluded completely, and those who failed one and passed the other had their results manually examined for suspicious patterns, with their data included or excluded on a case-by-case basis before any other data analysis had taken place.

In the online-VAAST, participants were filtered by error rate, such that those with less than 60% success in following the instructions were excluded on the *reaction time* measures. As in other studies of face perception (Sui & Humphreys, 2013), trials with a reaction time of less than 200ms were excluded from the implicit reaction time analysis. Furthermore, following Aubé and colleagues’ (2019) application of the online-VAAST, trials with reaction times above 2500ms were also excluded from this analysis. Individuals who had a high Cook’s distance (using an algorithm presented by Corradi et al., 2018) on any of the dependent measures were excluded for that measure alone. Participants excluded on the implicit measures criteria detailed in this paragraph were still included on the tests of explicit measures.

Analyses for any significant effects found for any of the models (both hypothesized and exploratory) were repeated with no cases excluded to test whether they are still significant.

### S4. Blinding

Participants did not know of the existence of any treatment group other than the one to which they have been assigned. Data collection occurred automatically online and was therefore blind (all participants were randomly assigned to one or other condition before any data was collected from them). Data analysis was not blind to condition; however, all data and code used in the analysis has been uploaded to the OSF project page.

### S5. Statement of informed consent

*The following text was presented to all participants before they began the online experiment:*

Confirm you want to do this survey

Institutions: [redacted for peer review]

Study title: “Social perception of people who use masks in times of emergency due to COVID-19”.

Principal Investigators: [redacted for peer review]

Aim: to identify how certain individual differences can influence the perception of “ambiguously” ill people, at the moment of psychosocial emergency that we are going through worldwide.

1. Your participation in this study does not entail any known risk to your health.

2. Participation in this study is voluntary and can be canceled at any time. If you withdraw, you can decide whether your data should be deleted or whether it can continue to be used after being made anonymous.

3. The data derived from participation will be used exclusively for research, study and publication purposes, always safeguarding the right to privacy and anonymity.

4. The study will be carried out following the international ethical criteria set forth in the Declaration of Helsinki (2008).

5. All relevant data for the study will be collected and stored in compliance with current data protection regulations. This ensures that all of your data – without restrictions – is treated confidentially.

Please confirm that you wish to participate in this study.

🞏 I understand the conditions of this study


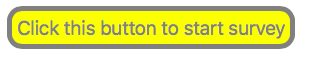


### S6. Demographic questions

Please choose one:

- Female
- Male
- Other

How old are you?

Please select your age:

What is the highest educational level that you have completed?

- Primary education
- Secondary education (<= 16 years)
- Secondary education or professional qualification (> 16 years)
- Undergraduate degree
- Postgraduate degree
- Doctorate

Which is your country of residence?

- United States
- United Kingdom

*[If “United States” selected:]*

Generally speaking, do you usually think of yourself as a Republican, a Democrat, an Independent, or something else?

- Republican
- Democrat
- Independent
- Something else

*[If “United Kingdom” selected:]*

In General Elections, do you usually vote Conservative, Labour, Liberal Democrat, or another party?

- Conservative
- Labour
- Liberal Democrat
- Other

### S7. Experimental materials

| **Experimental stimulus** | **Control stimulus** |
| --- | --- |
| 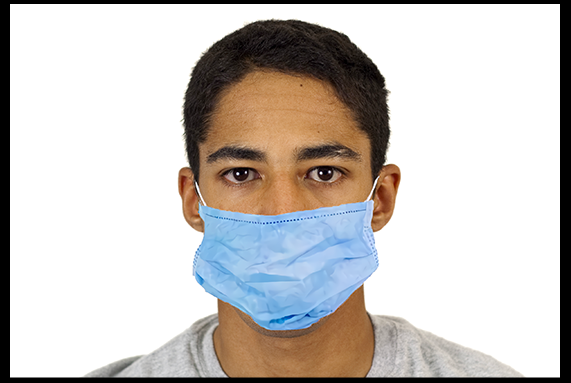 | 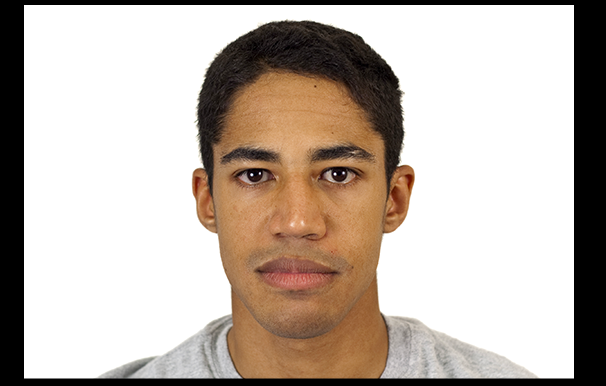 |
| 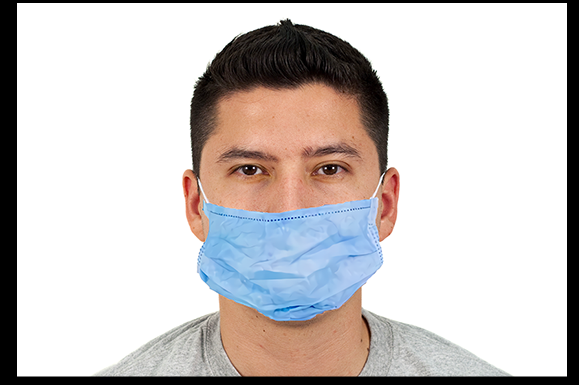 | 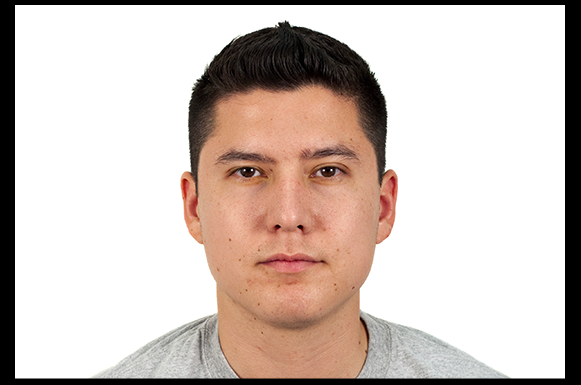 |
| 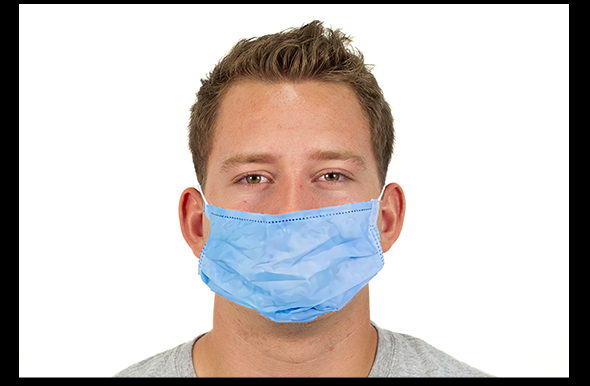 | 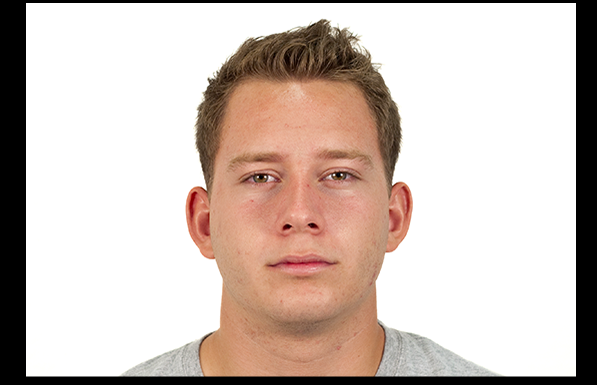 |
| 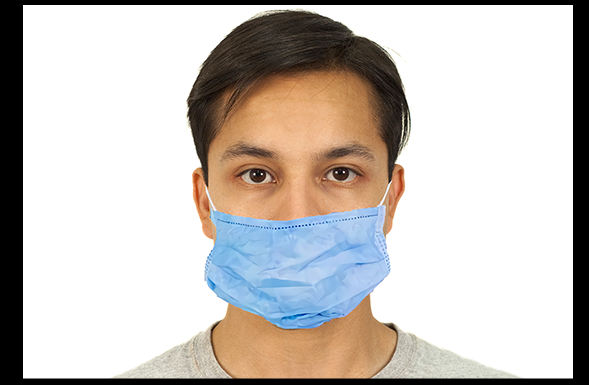 | 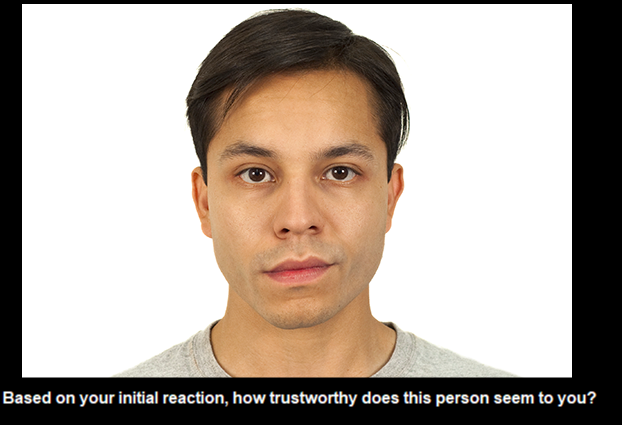 |
| 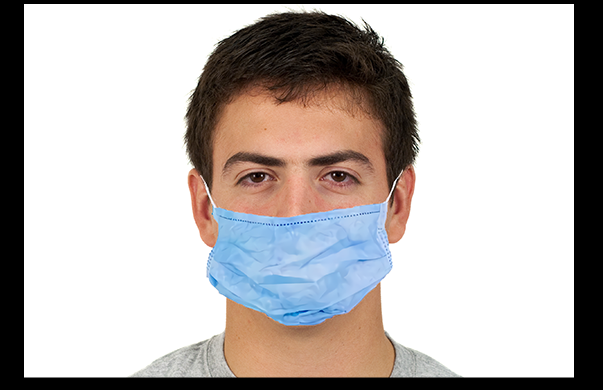 | 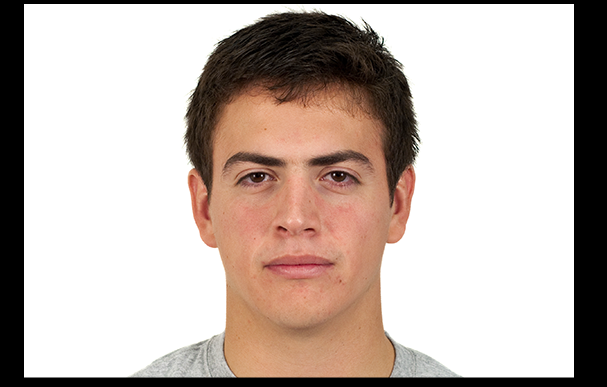 |

*For each face, each of the following questions was asked (separately):*

- Based on your initial reaction, how trustworthy does this person seem to you?

| Not at all trustworthy | 1 | 2 | 3 | 4 | 5 | 6 | 7 | Very trustworthy |
| --- | --- | --- | --- | --- | --- | --- | --- | --- |

- Please complete the following statement, indicating the “closest” level of interaction with which you would feel comfortable: “I’d feel comfortable if this person were…”

| 1 = A close friend / romantic partner | 2 = My roommate / housemate | | | 3 = Someone who lives in my apartment building / the house next door | | | 4 = A classmate / work colleague | | 5 = Someone who studies in my university / works for the same company | | 6 = Someone who lives in my neighborhood | | 7 = Someone who lives in my city |
| --- | --- | --- | --- | --- | --- | --- | --- | --- | --- | --- | --- | --- | --- |
| Closest level | | 1 | 2 | | 3 | 4 | | 5 | | 6 | | 7 | Most distant level |

- Based on your initial reaction, do you think that this person is sick or healthy?
  He’s healthy 🞏 🞏 He’s sick

### S8. Standard International and Generalized Trust scales

Please indicate your answer to the following question. 0 means you can't trust and 10 means you can trust the people in your neighborhood.

| **Item** | **0** | **1** | **2** | **3** | **4** | **5** | **6** | **7** | **8** | **9** | **10** |
| --- | --- | --- | --- | --- | --- | --- | --- | --- | --- | --- | --- |
| Please indicate your answer to the following question. 0 means you can't trust and 10 means you can trust the people in your neighborhood. |  |  |  |  |  |  |  |  |  |  |  |

Using the following scale, please indicate how much you agree or disagree with the following statements.

| **Item** | **1: Totally disagree** | **2** | **3** | **4** | **5: Totally agree** |
| --- | --- | --- | --- | --- | --- |
| People in my neighborhood are basically honest |  |  |  |  |  |
| People in my neighborhood are trustworthy |  |  |  |  |  |
| People in my neighborhood are basically nice and friendly |  |  |  |  |  |
| People in my neighborhood trust in other people |  |  |  |  |  |
| I am trustworthy |  |  |  |  |  |
| People in my neighborhood will respond in a friendly way when other people trust them |  |  |  |  |  |

### S9. Three-Domain Disgust Scale (Pathogen Disgust subscale)

The following situations describe a variety of concepts. Please rate how unpleasant you find the concepts described in the situations, where 0 means that you do not find the concept unpleasant at all and 6 means that you find the concept extremely unpleasant.

| **Item** | **0: Not at all unpleasant** | **1** | **2** | **3** | **4** | **5** | **6: Extremely unpleasant** |
| --- | --- | --- | --- | --- | --- | --- | --- |
| Standing near a person who has body odor |  |  |  |  |  |  |  |
| Shaking hands with a stranger who has sweaty palms |  |  |  |  |  |  |  |
| Stepping on dog poop |  |  |  |  |  |  |  |
| Accidentally touching a person’s bloody cut |  |  |  |  |  |  |  |
| Seeing some mold on old leftovers in your refrigerator |  |  |  |  |  |  |  |
| Sitting next to someone who has red sores on the arm |  |  |  |  |  |  |  |
| Seeing a cockroach run across the floor |  |  |  |  |  |  |  |

### S10. Liebowitz Social Anxiety scale (Avoidance subscale)

How often do you avoid these situations?

| **Item** | **Never 0%** | **Occasionally 1-33%** | **Often 34-66%** | **Usually 67-100%** |
| --- | --- | --- | --- | --- |
| Using a telephone in public |  |  |  |  |
| Participating in a small group activity |  |  |  |  |
| Eating in public |  |  |  |  |
| Drinking with others |  |  |  |  |
| Talking to someone in authority |  |  |  |  |
| Acting, performing, or speaking in front of an audience |  |  |  |  |
| Going to a party |  |  |  |  |
| Working while being observed |  |  |  |  |
| Writing while being observed |  |  |  |  |
| Calling someone you don't know very well |  |  |  |  |
| Talking face to face with someone you don't know very well |  |  |  |  |
| Meeting strangers |  |  |  |  |
| Urinating in a public bathroom |  |  |  |  |
| Entering a room when others are already seated |  |  |  |  |
| Being the center of attention |  |  |  |  |
| Speaking up at a meeting |  |  |  |  |
| Taking a test of your ability, skill, or knowledge |  |  |  |  |
| Expressing disagreement or disapproval to someone you don't know very well |  |  |  |  |
| Looking someone who you don't know very well straight in the eyes |  |  |  |  |
| Giving a prepared oral talk to a group |  |  |  |  |
| Trying to make someone's acquaintance for the purpose of a romantic/sexual relationship |  |  |  |  |
| Returning goods to a store for a refund |  |  |  |  |
| Giving a party |  |  |  |  |
| Resisting a high pressure sales person |  |  |  |  |

## Results

### S11. Analytical strategy

All analyses were conducted within the R environment for statistical computing (R Core Team, 2018). Participants' responses to the questionnaires and stimuli were analyzed using linear mixed effects models, which account simultaneously for between-subjects and within-subjects effects of the independent variables (Snijders & Bosker, 2012). The mixed() function of the afex R package was used to produce the inferential statistics and p-values. The lsmeans package was used to calculate the predicted means for the fixed effects.

Model fitting and selection followed considerations for simplicity and hypothesis testing outlined by Barr et al (2013), Brauer and Curtin (2018) and Meteyard and Davies (2020). Where the relevant maximal model failed to converge, we removed the by unit random intercepts leaving us with by-stimuli and by-condition random slopes (except for perceived sickness, where model convergence was only achieved with a random intercept by participant model). We also ran separate post hoc analyses for each of the two relevant contrasts of country/political-preference pairs (i.e., UK-Conservative/Labour, US-Democrat/Republican) using pairwise comparisons alpha-corrected by the Satterthwaite method (Keselman et al., 1999). There were slight deviations from the preregistration, consisting mainly of privileging maximal models for confirmatory hypothesis testing as opposed to data driven considerations to improve model fit (Barr et al, 2013). Finally, for all models we performed an analysis of influential cases based on Cook’s distance, to evaluate each participant's influence on the results by examining the impact of its removal from the data set.

**S12. Complete models predicting trustworthiness judgments**

| *Models and Fixed Effects* | *β* | *SE* | *df* | *t* | *p* | *95 % CI* |
| --- | --- | --- | --- | --- | --- | --- |
| Model 1 |  |  |  |  |  |  |
| Condition | .063 | .013 | 14.04 | 4.92 | < .001 *** | .037, .09 |
| Disgust Sensitivity | .00017 | .00055 | 600 | .31 | .76 | -.0009, .0013 |
| Social Anxiety | .00017 | .00032 | 600 | .53 | .60 | -.0005, .0008 |
| Generalized Social Trust | .31 | .026 | 604 | 11.9 | < .001 *** | .26, .36 |
| Political orientation | -.002 | .002 | 604 | -1 | .32 | -.006, .002 |
| Labour/Democrat vs. Conservative/Republican | .008 | .015 | 600 | .55 | .58 | -.022, .038 |
| Labour/Democrat vs. Other candidate | .00075 | .012 | 600 | .064 | .95 | -.023, .025 |
| Conservative/Republican vs. Other candidate | .0072 | .015 | 600 | .48 | .63 | -.023, .037 |
| Condition * Disgust Sensitivity | -.001 | .0008 | 1127 | -1.40 | .16 | -.0026, .0006 |
| Condition * Social Anxiety | -.0003 | .0005 | 1124 | -.59 | .56 | -.0013, .0007 |
| Condition * Generalized Social Trust | -.0045 | .041 | 1092 | -.11 | .91 | -.086, .077 |
| Condition * Political orientation | -.01 | .003 | 1135 | -3.5 | < .001 *** | -.016, -.004 |
| Condition * Labour/Democrat vs.  Conservative/Republican | .0016 | .021 | 1144 | .08 | .94 | -.04, .04 |
| Condition * Labour/Democrat vs.  Other candidate | -.013 | .0177 | 1112 | -.76 | .44 | -.048, .022 |
| Condition * Conservative/Republican vs.  Other candidate | .015 | .022 | 1132 | .68 | .49 | -.03, .06 |
| Model 2 | ***β*** | ***SE*** | ***df*** | ***t*** | ***p*** | ***95 % CI*** |
| Condition | .077 | .11 | 606 | .68 | .49 | -.14, .30 |
| Country | .0022 | .0092 | 594 | .24 | .81 | -.016, .02 |
| Gender | .015 | .0094 | 594 | 1.6 | .11 | -.004, .034 |
| Age | .0012 | .00036 | 604 | 3.37 | < .001 *** | .0005, .002 |
| Sequence 1 | -.012 | .11 | 603.7 | -.11 | .91 | -.34, 0.1 |
| Sequence 2 | -.017 | .01 | 540 | -1.67 | .09 | -.037, .003 |
| Sequence 3 | .0055 | .11 | 603.6 | .05 | .96 | -.21, .225 |
| Educational level | .0093 | .0045 | 604 | 2.05 | .04 * | .0003, .018 |
| Targets sex | -.04 | .029 | 8 | -1.32 | .22 | -.098, .018 |
| Condition * Country | .0068 | .014 | 1096 | .49 | .63 | -.02, .035 |
| Condition * Gender | -.0095 | .014 | 1097 | -.66 | .50 | -.037, .018 |
| Condition * Age | -.0008 | .00056 | 1081 | -1.43 | .15 | -.002, .0003 |
| Condition * Educational level | .005 | .0066 | 1121 | .75 | .45 | -.008, .018 |
| Condition * Targets sex | -.0093 | .024 | 8 | -.38 | .71 | -.057, .039 |

*Note*. * *p* <= .05, ** *p* <= .01, *** *p* <= .001.

### S13. Complete models predicting sickness judgments

| *Models and Fixed Effects* | *β* | *SE* | *Z* | *p* | *95 % CI* |
| --- | --- | --- | --- | --- | --- |
| Model 1 |  |  |  |  |  |
| Condition | -.44 | .12 | -3.63 | < .001 *** | -.68, -.20 |
| Disgust Sensitivity | .019 | .008 | 2.3 | .021 * | .003, .035 |
| Social Anxiety | .004 | .0046 | .86 | .39 | -.005, .013 |
| Generalized Social Trust | -.89 | .37 | -2.42 | .015 * | -1.6, -.15 |
| Political orientation | .08 | .037 | 2.2 | .027 * | .006, .154 |
| Labour/Democrat vs. Conservative/Republican | -.72 | .21 | -3.44 | < .001 *** | -1.14, -.30 |
| Labour/Democrat vs. Other candidate | -.29 | .16 | -1.8 | .072 | -.61, .03 |
| Conservative/Republican vs. Other candidate | -.42 | .217 | -1.95 | .05 * | -.85, .01 |
| Condition * Disgust Sensitivity | -.03 | .012 | -2.51 | .01 ** | -.054, -.006 |
| Condition * Social Anxiety | .013 | .0068 | 1.94 | .052 | -.0006, .027 |
| Condition * Generalized Social Trust | .155 | .577 | .27 | .79 | -1, 1.31 |
| Condition * Political orientation | -.003 | .053 | -.06 | .95 | -.11, .10 |
| Condition * Labour/Democrat vs.  Conservative/Republican | .52 | .29 | 1.78 | .075 | -.06, 1.1 |
| Condition * Labour/Democrat vs.  Other candidate | .61 | .24 | 2.51 | .012 * | .13, 1.09 |
| Condition * Conservative/Republican vs.  Other candidate | -.10 | .31 | -.29 | .77 | -.72, .52 |
| Model 2 | ***β*** | ***SE*** | ***Z*** | ***p*** | ***95 % CI*** |
| Condition | -.58 | .104 | -5.6 | < .001 *** | -.79, -.37 |
| Country | .16 | .12 | 1.3 | .19 | -.08, .40 |
| Gender | -.186 | .125 | -1.5 | .13 | -.44, .064 |
| Age | -.01 | .0048 | -2.34 | .02 * | -.02, -.0004 |
| Sequence 1 | -6.1 | 10.25 | -.60 | .55 | -26.6, 14.4 |
| Sequence 2 | 6.4 | 10.25 | .62 | .53 | -14.1, 26.9 |
| Sequence 3 | -6.3 | 10.25 | -.61 | .54 | -26.8, 14.2 |
| Educational level | .12 | .06 | 1.92 | .055 | 0, .24 |
| Targets sex | -.73 | .43 | -1.68 | .09 | -1.6, .13 |
| Condition * Country | -.30 | .184 | -1.66 | .097 | -.67, .07 |
| Condition * Gender | .06 | .186 | .32 | .75 | -.31, .43 |
| Condition * Age | .0034 | .007 | .46 | .64 | -.01, .017 |
| Condition * Educational level | -.18 | .086 | -2.06 | .04 * | -.35, -.008 |
| Condition * Targets sex | .74 | .11 | 6.65 | < .001 *** | .52, .96 |

*Note*. * *p* <= .05, ** *p* <= .01, *** *p* <= .001.

### S14. Models predicting social distance judgments

| *Models and Fixed Effects* | *β* | *SE* | *df* | *t* | *p* | *95 % CI* |
| --- | --- | --- | --- | --- | --- | --- |
| Model 1 |  |  |  |  |  |  |
| Condition | .015 | .007 | 48.87 | 2.14 | .037 * | .136, .03 |
| Disgust Sensitivity | .003 | .0006 | 1134 | 4.96 | < .001 *** | .0018, .0042 |
| Social Anxiety | .00057 | .00035 | 1105 | 1.62 | .10 | -.00013, .0013 |
| Generalized Social Trust | -.20 | .03 | 1054 | -6.6 | < .001 *** | -.26, -.14 |
| Political orientation | .015 | .0034 | 51.98 | 4.26 | < .001 *** | .14, .157 |
| Labour/Democrat vs. Conservative/Republican | .012 | .009 | 805 | 1.33 | .18 | -.006, .03 |
| Labour/Democrat vs. Other candidate | .004 | .0076 | 1092 | .57 | .57 | -.01, .02 |
| Conservative/Republican vs. Other candidate | -.01 | .021 | 432.4 | -.46 | .64 | -.052, .032 |
| Condition * Disgust Sensitivity | .0016 | .0006 | 1134 | 2.69 | .007 ** | .0004, .0028 |
| Condition * Social Anxiety | -.00053 | .00035 | 1105 | -1.5 | .13 | -.0012, .00017 |
| Condition * Generalized Social Trust | .046 | .032 | 1054 | 1.52 | .13 | -.018, .11 |
| Condition * Political orientation | .00027 | .0028 | 465 | .10 | .92 | -.005, .0059 |
| Condition * Labour/Democrat vs.  Conservative/Republican | -.021 | .009 | 805 | -2.26 | .024 * | -.23, -.003 |
| Condition * Labour/Democrat vs.  Other candidate | .003 | .0076 | 1092 | .42 | .67 | -.012, .018 |
| Condition * Conservative/Republican vs.  Other candidate | .077 | .031 | 847.3 | 2.46 | .014 * | .015, .14 |
| Model 2 | ***β*** | ***SE*** | ***df*** | ***t*** | ***p*** | ***95 % CI*** |
| Condition | -.014 | .022 | 613 | -.63 | .53 | -.06, .03 |
| Country | .0015 | .0018 | 597 | .82 | .41 | -.002, .005 |
| Gender | -.0005 | .0019 | 597 | -.29 | .77 | -.004, .003 |
| Age | .008 | .0035 | 608 | 2.3 | .02 * | .001, .015 |
| Sequence 1 | -.01 | .022 | 607.2 | -.46 | .64 | -.05, .034 |
| Sequence 2 | -.00017 | .002 | 538 | -.08 | .93 | -.004, .004 |
| Sequence 3 | -.009 | .022 | 607.2 | -.40 | .69 | -.05, .035 |
| Educational level | -.085 | .044 | 608 | -1.92 | .055 | -.17, .003 |
| Targets sex | .006 | .0028 | 8 | 2.12 | .067 | .004, .011 |
| Condition * Country | -.0012 | .0028 | 1089 | -.43 | .66 | -.007, .004 |
| Condition * Gender | .00056 | .0029 | 1092 | .20 | .84 | -.005, .006 |
| Condition * Age | -.00004 | .0001 | 1111 | -.80 | .42 | -.0002, .0001 |
| Condition * Educational level | .00004 | .0013 | 1151 | .034 | .97 | -.0025, .0026 |
| Condition * Targets sex | .00074 | .003 | 8 | .26 | .80 | -.005, .007 |

*Note*. * *p* <= .05, ** *p* <= .01, *** *p* <= .001.

### S15. Model predicting implicit Approach action to targets in the O-VAAST.

| *Fixed Effects* | *β* | *SE* | *df* | *t* | *p* | *95 % CI* |
| --- | --- | --- | --- | --- | --- | --- |
| Sequence | 16.35 | 14 | 1140.5 | 1.17 | .24 | -11.1, 43.8 |
| Target: Masked vs. Unmasked | -63.6 | 8.75 | 23.5 | -7.26 | < .001 *** | -80.7, -46.4 |
| Political orientation | 9.15 | 3.26 | 1149.4 | 2.81 | .005 ** | 2.8, 15.5 |
| Labour/Democrat vs. Conservative/Republican | 35.37 | 22.7 | 1153.1 | 1.56 | .12 | -9.1, 80 |
| Labour/Democrat vs. Other candidate | 14.02 | 19.4 | 1136.1 | .72 | .47 | -24, 52 |
| Conservative/Republican vs. Other candidate | 21.3 | 24.36 | 1142 | .88 | .38 | -26.4, 69.1 |
| Targets sex | 22.3 | 7.78 | 8 | 2.86 | .02 * | 7.04, 37.6 |
| Sequence * Target | -6.56 | 11 | 1125.2 | -.60 | .55 | -28.1, 15 |
| Target * Political orientation | .57 | 2.56 | 1141.6 | .22 | .82 | -4.5, 5.6 |
| Target * Labour/Democrat vs.  Conservative/Republican | 28.3 | 17.9 | 1153.8 | 1.58 | .11 | -6.8, 63.4 |
| Target * Labour/Democrat vs.  Other candidate | 1.96 | 15.2 | 1121.2 | .13 | .90 | -27.8, 31.8 |
| Target * Conservative/Republican vs.  Other candidate | 26.3 | 19.15 | 1134.5 | 1.37 | .17 | -11.2, 64 |
| Target * Targets sex | -20.8 | 11.23 | 16 | -1.85 | .08 | -42.8, 1.2 |

*Note*. * *p* <= .05, ** *p* <= .01, *** *p* <= .001.

### S16. Model predicting implicit Avoidance action to targets in the O-VAAST.

| *Fixed Effects* | *β* | *SE* | *df* | *t* | *p* | *95 % CI* |
| --- | --- | --- | --- | --- | --- | --- |
| Sequence | -.15 | 13.3 | 1165.6 | .10 | .99 | -26.2, 25.9 |
| Target: Masked vs. Unmasked | .99 | 11.1 | 1509.7 | .09 | .93 | -20.8, 22.7 |
| Political orientation | 7 | 3.9 | 1169.6 | 1.8 | .076 | -.73, 14.7 |
| Labour/Democrat vs. Conservative/Republican | 49.6 | 21.5 | 1169.4 | 2.3 | .02 * | -49, 26.8 |
| Labour/Democrat vs. Other candidate | 15.8 | 18.4 | 1163 | .85 | .39 | -42.8, 22 |
| Conservative/Republican vs. Other candidate | 33.8 | 23.1 | 1167.3 | -1.46 | .14 | -79.2, 11.5 |
| Targets sex | 4.4 | 5.48 | 19482.5 | .80 | .42 | -6.35, 15.1 |
| Sequence * Target | 37 | 12 | 1145.6 | 3.1 | .002 ** | 13.5, 60.4 |
| Target * Political orientation | -5.9 | 3.55 | 1152.5 | -1.66 | .097 | -12.8, 1.1 |
| Target * Labour/Democrat vs.  Conservative/Republican | -11.1 | 19.3 | 1149.5 | -.57 | .56 | -49, 26.8 |
| Target * Labour/Democrat vs.  Other candidate | -10.4 | 16.5 | 1137 | -.63 | .53 | -42.8, 22 |
| Target * Conservative/Republican vs.  Other candidate | .74 | 20.8 | 1145.9 | .035 | .97 | -40, 41.5 |
| Target * Targets sex | 11.3 | 7.82 | 19512 | 1.44 | .15 | -4.06, 26.6 |

*Note*. * *p* <= .05, ** *p* <= .01, *** *p* <= .001.

### S17. Model predicting implicit actions to targets with mask in the O-VAAST.

| *Fixed Effects* | *β* | *SE* | *df* | *t* | *p* | *95 % CI* |
| --- | --- | --- | --- | --- | --- | --- |
| Sequence | 36.3 | 14 | 746 | 2.6 | .01 ** | 8.8, 63.9 |
| Action: Approach vs. Avoidance | -33.7 | 12.4 | 29.3 | -2.7 | .011 * | -58, -9.3 |
| Political orientation | 1 | 4.14 | 1158.9 | .24 | .81 | -7.1, 9.1 |
| Labour/Democrat vs. Conservative/Republican | 41.8 | 17.8 | 1162.4 | 2.35 | .019 * | 7, 76.7 |
| Labour/Democrat vs. Other candidate | 6.3 | 18.7 | 1153.5 | .34 | .74 | -30.4, 43 |
| Conservative/Republican vs. Other candidate | 35.5 | 22.5 | 1157.6 | 1.6 | .11 | -8.6, 79.7 |
| Targets sex | 16 | 6 | 30.6 | 2.67 | .012 * | 4.2, 27.7 |
| Sequence * Target | -26.1 | 11.9 | 112.4 | -2.2 | .03 * | -49.3, -2.9 |
| Action * Political orientation | 1.54 | 3.4 | 1157.6 | .45 | .65 | -5.1, 8.2 |
| Action * Labour/Democrat vs.  Conservative/Republican | 28.9 | 14.6 | 1151.6 | 1.98 | .048 * | .28, 57.5 |
| Action * Labour/Democrat vs.  Other candidate | 12.7 | 15.3 | 1139 | .83 | .41 | -17.3, 42.7 |
| Action * Conservative/Republican vs.  Other candidate | 16.2 | 18.5 | 1146 | .87 | .38 | -20, 52.4 |
| Action * Targets sex | -15.3 | 11 | 10.4 | -1.38 | .19 | -37, 6.4 |

*Note*. * *p* <= .05, ** *p* <= .01, *** *p* <= .001.

### S18. Model predicting implicit actions to targets without mask in the O-VAAST.

| *Fixed Effects* | *β* | *SE* | *df* | *t* | *p* | *95 % CI* |
| --- | --- | --- | --- | --- | --- | --- |
| Sequence | -.23 | 13.3 | 1163.7 | -.02 | .98 | -26.3, 25.8 |
| Action: Approach vs. Avoidance | 34 | 11.2 | 166.3 | 3.02 | .003 ** | 11.95, 56 |
| Political orientation | 7.1 | 3.94 | 1167.9 | 1.8 | .07 | -.61, 14.8 |
| Labour/Democrat vs. Conservative/Republican | 49.3 | 21.5 | 1167.6 | 2.3 | .02 * | 7.2, 91.5 |
| Labour/Democrat vs. Other candidate | 15.2 | 18.4 | 1161 | .83 | .41 | -20.9, 51.4 |
| Conservative/Republican vs. Other candidate |  |  |  |  |  |  |
| Targets sex | 4.24 | 5.87 | 47.1 | .72 | .47 | -7.3, 15.7 |
| Sequence * Action | 17 | 11.6 | 1123.9 | 1.46 | .14 | -5.8, 39.8 |
| Action * Political orientation | -2 | 3.47 | 1151.7 | -.57 | .56 | -8.8, 4.8 |
| Action * Labour/Democrat vs.  Conservative/Republican | -14 | 18.9 | 1145.3 | -.74 | .46 | -51.1, 23.1 |
| Action * Labour/Democrat vs.  Other candidate | -1.36 | 16.1 | 1116.1 | -.08 | .93 | -32.9, 30.2 |
| Action * Conservative/Republican vs.  Other candidate |  |  |  |  |  |  |
| Action * Targets sex | 18.1 | 9 | 18.7 | 2.01 | .058 | .10, 36.1 |

*Note*. * *p* <= .05, ** *p* <= .01, *** *p* <= .001.

### S19. Correlations between results from the explicit judgements and the O-VAAST.

|  |  |  |  |  | ***Masked target*** | | ***Unmasked target*** | |
| --- | --- | --- | --- | --- | --- | --- | --- | --- |
|  | ***Approach*** | ***Avoidance*** | ***Masked target*** | ***Unmasked target*** | ***Approach*** | ***Avoidance*** | ***Approach*** | ***Avoidance*** |
| **Trustworthiness** | .013 | .058 * | -.03 | .07 * | -.06 | .0017 | .029 | .105 ** |
| **Perception of Sickness** | -.002 | -.014 | -.006 | .025 | -.02 | .007 | .027 | .023 |
| **Social Distance** | .133 *** | .127 *** | .155 *** | .107 *** | .16 *** | .143 *** | .107 ** | .11 ** |

*Note*. Spearman correlations. Positive correlation between trustworthiness and avoidance shows that faster RTs in this action were associated with lower trustworthiness scores. This relationship was only found with unmasked targets. Positive correlations between social distance and approach and avoidance show that faster RTs in these actions were associated with lower scores in social distance (perceiving targets as more socially desirable).* *p* <= .05, ** *p* <= .01, *** *p* <= .001.

### S20. Demographic effects on the explicit measures

As participants’ age and educational level increased, levels of trust in both targets also increased. In contrast, as participants’ age increased, sickness perception of targets decreased, while as educational level increased, sickness perception tended to increase. The interaction with Condition revealed that higher educational level only predicted an increased sickness perception to targets without a mask. Lastly, participants perceived female targets without a mask (*M* = .193, 95 % CI [.17, .215]) as more likely to be sick than the same female targets with a mask (*M* = .13, 95 % CI [.11, .15]), Z = -5.6, *p* < .001. Conversely, there were no significant differences between male targets without a mask (*M* = .123, 95 % CI [.104, .142]) and the same male targets with a mask (M = .14, 95 % CI [.12, .16]), Z = 1.47, *p* = .14. Finally, as participants’ age increased, desired social distance from targets also increased.

### S21. Political orientation and sickness perceptions

We had no hypotheses regarding interactions between political orientation and perceptions of sickness in mask-wearers. Conservatives perceived both types of targets as more likely to be sick than liberals did. In contrast, participants voting Labour/Democrat (*M* = .15, 95% CI [.13, .17]) perceived targets as more likely to be sick than participants voting Conservative/Republican (*M* = .127, 95% CI [.11, .15]). The interaction with condition revealed that this difference was significant for maskless targets (*p* = .001), but not for masked targets (*p* = .66). Similarly, participants voting “Other” (*M* = .158, 95% CI [.14, .18]) perceived targets as more likely to be sick than participants voting Conservative/Republican.

### S22. Target gender and sequence effects on the implicit measures

Participants approached male targets (M = 829, SD = 244.8, 95% CI [815, 842]) significantly faster than female targets (M = 840, SD = 245.1, 95% CI [826, 854]).

In the avoidance action model, there was a significant interaction between Target x Sequence. When avoidance to targets without a mask was in the first block, RTs were faster to these targets (M = 831, SD = 353.6, 95% CI [811, 851]) than to targets with a mask (M = 867, SD = 371, 95% CI [846, 888]), t(1136) = 3.98, *p* < .001. In contrast, when avoidance to targets with a mask was in the first block, RTs to targets with (M = 831, SD = 388.3, 95% CI [809, 853]) and without a mask (M = 831, SD = 371, 95% CI [810, 852]) did not significantly differ, t(1150) = -.04, *p* = .96.

In the masked targets model, a significant effect of Sequence revealed that participants’ RTs to targets with a mask were faster when in the first block they approached these targets (*M* = 818, SD = 333.9, 95% CI [799, 837]), than when in the first block they avoided these targets (*M* = 841, SD = 318.3, 95% CI [823, 859]). Lastly, the interaction between Action x Sequence supported larger RTs differences to targets with a mask when the approach action was first, *t*(89.2) = -5.97, *p* < .001, than when the avoidance action was first, *t*(28.2) = -2.6, *p* = .016.

Participants responded faster to male (*M* = 825, SD = 258.3, 95% CI [811, 840]) than to female targets with a mask (*M* = 834, SD = 258.3, 95% CI [819, 848].

**S23. Models predicting the influence of political orientation and individual differences on explicit judgements.**

| *Models* | *Trustworthiness* | | | | *Sickness Perception* | | | | *Social Distance* | | | |
| --- | --- | --- | --- | --- | --- | --- | --- | --- | --- | --- | --- | --- |
| *Fixed effects* | *β* | *t* | *p* | *95 % CI* | *β* | *Z* | *p* | *95 % CI* | *β* | *t* | *p* | *95 % CI* |
| Condition | -.060 | -9.02 | < .001 *** | -.07, -.05 | .34 | 3.35 | < .001 *** | .14, .54 | .027 | 2.10 | .043 * | .001, .053 |
| Political Orientation | -.011 | -4.73 | < .001 *** | -.02, -.01 | .097 | 2.90 | .0037 ** | .03, .16 | .017 | 4.36 | < .001 *** | .01, .03 |
| Disgust Sensitivity | -.0010 | -1.66 | .096 | -.002, .0002 | -.015 | -1.82 | .068 | -.03, .001 | .00003 | .035 | .97 | -.002, .002 |
| Social Anxiety | .0002 | .56 | .57 | -.0005, .001 | .016 | 3.18 | .0014 ** | .01, .03 | .0006 | 1.08 | .28 | -.005, .002 |
| Generalized Social Trust | .26 | 8 | < .001 *** | .20, .33 | -.76 | -1.66 | .097 | -1.66, .14 | -.18 | -3.72 | < .001 *** | -.27, -.08 |
| Condition * Political  Orientation | .0091 | 2.81 | .0051 ** | .003, .016 | -.094 | -2.13 | .033 * | -.18, -.01 | .0049 | -1.02 | .31 | -.15, .005 |
| Condition * Disgust  Sensitivity | .0008 | .97 | .33 | -.0009, .003 | .031 | 2.75 | .006 ** | .01, .05 | .0034 | 2.85 | .0044 ** | .001, .006 |
| Condition * Social  Anxiety | -.0003 | -.63 | .53 | -.001, .0007 | -.013 | -1.94 | .052 | .004, -.03 | .0004 | -.56 | .58 | -.002, .001 |
| Condition * Generalized  Social Trust | .021 | .49 | .62 | -.065, .11 | -.28 | -.48 | .63 | -1.42, .86 | .065 | 1.06 | .29 | -.06, .19 |

*Note*. * *p* <= .05, ** *p* <= .01, *** *p* <= .001.

**S24. Comparison analyses to assess the influence of political orientation and voting intention in the explicit judgements.**

| **Models** | | *df* | *AIC* | *BIC* | *logLik* | *Test* | *L.Ratio* | *p-value* |
| --- | --- | --- | --- | --- | --- | --- | --- | --- |
| **Trustworthiness** | |  |  |  |  |  |  |  |
| 1 | Only voting intention | 13 | -11847 | -11751 | 5936.6 |  |  |  |
| 2 | Only political orientation | 11 | -11862 | -11781 | 5942.3 |  |  |  |
| 3 | Voting intention and political orientation | 15 | -11860 | -11749 | 5945 | 1 vs. 3 | 16.7 | < .001 *** |
|  |  |  |  |  |  | 2 vs. 3 | 5.4 | .25 |
| **Sickness Perception** | |  |  |  |  |  |  |  |
| 1 | Only voting intention | 8 | 9681.7 | 9741.1 | -4832.9 |  |  |  |
| 2 | Only political orientation | 6 | 9685.3 | 9729.8 | -4836.6 |  |  |  |
| 3 | Voting intention and political orientation | 10 | 9679.7 | 9753.9 | -4829.8 | 1 vs. 3 | 6.01 | .049 * |
|  |  |  |  |  |  | 2 vs. 3 | 13.57 | .009 ** |
| **Social Distance** | |  |  |  |  |  |  |  |
| 1 | Only voting intention | 13 | -7944.5 | -7848 | 3985.3 |  |  |  |
| 2 | Only political orientation | 11 | -7958.7 | -7877 | 3990.3 |  |  |  |
| 3 | Voting intention and political orientation | 15 | -7959.5 | -7848.2 | 3994.7 | 1 vs. 3 | 19 | < .001 *** |
|  |  |  |  |  |  | 2 vs. 3 | 8.82 | .066 |

*Note.* *AIC* indicates Akaike information criterion. *BIC* indicates Bayesian information criterion. AIC and BIC are indices of fit of the model. A decrease in AIC and BIC from a model to another model indicates that the fit is improving. *logLik* indicates the log-likelihood. *L.Ratio* is the likelihood ratio. Significant *p-values* indicate that the model fit significantly improves when we considered voting intention or political orientation as predictor.

**S25. Comparison analyses to assess the influence of political orientation and voting intention in the implicit O-VAAST.**

| **Models** | | *df* | *AIC* | *BIC* | *logLik* | *Test* | *L.Ratio* | *p-value* |
| --- | --- | --- | --- | --- | --- | --- | --- | --- |
| **Approach** | |  |  |  |  |  |  |  |
| 1 | Only voting intention | 15 | 311405.4 | 311525.2 | -155688 |  |  |  |
| 2 | Only political orientation | 13 | 311430.8 | 311534.6 | -155702 |  |  |  |
| 3 | Voting intention and political orientation | 17 | 311399.2 | 311534.9 | -155682 | 1 vs. 3 | 10.2 | .006 ** |
|  |  |  |  |  |  | 2 vs. 3 | 39.6 | < .001 *** |
| **Avoidance** | |  |  |  |  |  |  |  |
|  |  |  |  |  |  |  |  |  |
| 1 | Only voting intention | 15 | 313428 | 313548 | -156699 |  |  |  |
| 2 | Only political orientation | 13 | 313425 | 313529 | -156700 |  |  |  |
| 3 | Voting intention and political orientation | 17 | 313428 | 313564 | -156697 | 1 vs. 3 | 4.25 | .12 |
|  |  |  |  |  |  | 2 vs. 3 | 5.68 | .22 |
| **Mask** | |  |  |  |  |  |  |  |
| 1 | Only voting intention | 51 | 312518 | 312925 | -156208 |  |  |  |
| 2 | Only political orientation | 27 | 312479 | 312695 | -156212 |  |  |  |
| 3 | Voting intention and political orientation | 31 | 312478 | 312725 | -156208 | 1 vs. 3 | 37.2 | < .001 *** |
|  |  |  |  |  |  | 2 vs. 3 | 9.31 | .054 |
| **No mask** | |  |  |  |  |  |  |  |
| 1 | Only voting intention | 15 | 312436 | 312556 | -156203 |  |  |  |
| 2 | Only political orientation | 13 | 312433 | 312537 | -156204 |  |  |  |
| 3 | Voting intention and political orientation | 17 | 312436 | 312572 | -156201 |  | 4.42 | .11 |
|  |  |  |  |  |  |  | 5.34 | .25 |

*Note.* *AIC* indicates Akaike information criterion. *BIC* indicates Bayesian information criterion. AIC and BIC are indices of fit of the model. A decrease in AIC and BIC from a model to another model indicates that the fit is improving. *logLik* indicates the log-likelihood. *L.Ratio* is the likelihood ratio. Significant *p-values* indicate that the model fit significantly improves when we considered voting intention or political orientation as predictor.

**S26. Correlations between results from the explicit judgements and the O-VAAST.**

|  |  |  |  |  | ***Masked target*** | | ***Unmasked target*** | |
| --- | --- | --- | --- | --- | --- | --- | --- | --- |
|  | ***Approach*** | ***Avoidance*** | ***Masked target*** | ***Unmasked target*** | ***Approach*** | ***Avoidance*** | ***Approach*** | ***Avoidance*** |
| **Trustworthiness** | .013 | .058 * | -.03 | .07 * | -.06 | .0017 | .029 | .105 ** |
| **Perception of Sickness** | -.002 | -.014 | -.006 | .025 | -.02 | .007 | .027 | .023 |
| **Social Distance** | .133 *** | .127 *** | .155 *** | .107 *** | .16 *** | .143 *** | .107 ** | .11 ** |

*Note*. Spearman correlations. Positive correlation between trustworthiness and avoidance shows that faster RTs in this action were associated with lower trustworthiness scores. This relationship was only found with unmasked targets. Positive correlations between social distance and approach and avoidance show that faster RTs in these actions were associated with lower scores in social distance (perceiving targets as more socially desirable).* *p* <= .05, ** *p* <= .01, *** *p* <= .001.

## Code

### S27. R code and output used to calculate observed power and to derive inputs for the standardized calculation of VPCs

library(simr)

contrasts(confiability$condition) <- c(-0.5, 0.5)

mo1 <- lmer(response ~ condition * disgust.c + condition * anxiety.c + condition * trust.1.c + condition * trust.c + gender + (1|subject_nr) + (1|face_number), data=confiability)

Linear mixed model fit by REML. t-tests use Satterthwaite's method ['lmerModLmerTest']

Formula: response ~ condition + disgust.c + anxiety.c + trust.1.c + trust.c + (1 | subject_nr) + (1 | face_number)

Data: mo_confiability REML criterion at convergence: 16099.4

Scaled residuals: Min 1Q Median 3Q Max -4.3474 -0.4905 0.0085 0.5391 3.8701

Random effects: Groups Name Variance Std.Dev.

subject_nr (Intercept) 0.53914 0.7343

face_number (Intercept) 0.03392 0.1842

Residual 1.13661 1.0661

Number of obs: 5000, groups: subject_nr, 1000; face_number, 5

Fixed effects:

Estimate Std. Error df t value Pr(>|t|)

(Intercept) 3.973878 0.086910 4.650363 45.724 2.37e-07 ***

condition1 -0.079577 0.027906 993.834272 -2.852 0.004440 **

disgust.c -0.009835 0.003469 993.834270 -2.835 0.004668 **

anxiety.c -0.004310 0.001134 993.834270 -3.799 0.000154 ***

trust.1.c 0.078578 0.014929 993.834269 5.263 1.73e-07 ***

trust.c 0.028432 0.009064 993.834269 3.137 0.001759 **

---

Signif. codes: 0 ‘***’ 0.001 ‘**’ 0.01 ‘*’ 0.05 ‘.’ 0.1 ‘ ’ 1

Correlation of Fixed Effects:

(Intr) cndtn1 dsgst. anxty. trs.1. condition1 0.020

disgust.c -0.001 0.084

anxiety.c -0.002 0.001 -0.150

trust.1.c -0.002 0.057 0.045 0.061

trust.c 0.001 -0.008 -0.062 0.107 -0.665

## Power simulation

powerSim(mo1, nsim=100, test = fcompare(response~condition)).

Power for model comparison, (95% confidence interval):==============================================================================================|

100.0% (96.38, 100.0)

Test: Likelihood ratio

Comparison to response ~ condition + [re]

Based on 100 simulations, (2 warnings, 0 errors)

alpha = 0.05, nrow = 5270

Time elapsed: 0 h 9 m 15 s

**S24. R code to be used in statistical analysis of results**

###### Explicit judgments and questionnaires ######

# Load packages

if (!require("pacman")) install.packages("pacman")

pacman::p_load("dplyr", "ggplot2", "lme4","afex","lmerTest","effects", "lsmeans", "ggeffects", "plotly","scales","influence.ME","performance”, "parameters")

data$question = as.character(gsub("1", "Trustworthiness", data$question))

data$question = as.character(gsub("2", "Social_distance", data$question))

data$question = as.character(gsub("3", "Sickness", data$question))

## Center continuous predictors

mean_center <- function(x) return(x - mean(x, na.rm=TRUE))

data1 <- data

vars_to_center <- c("Social_Anxiety", "Trust1", "Trustworthiness6", "Disgust")

colnames(data1)[colnames(data1) %in% vars_to_center] <- paste0(vars_to_center, ".c")

for (i in paste0(vars_to_center, ".c")){

data1[,c(i)] <- data1[,c(i)] - mean(data1[,c(i)], na.rm=TRUE)

}

data <- left_join( data, data1 )

## Subset each explicit judgment to conduct LMM

social_distance <- data[which(data$question == "Social_distance"),]

trustworthiness <- data[which(data$question == "Trustworthiness "),]

sickness <- data[which(data$question == "Sickness"),]

## forward stepwise regression ##

## A. social_distance

contrasts(social_distance$condition) <- c(-0.5, 0.5)

m1 <- lmer(response ~ 1 + (1|subject_nr) + (1|face_number), data=social_distance)

summary(m1)

m2 <- lmer(response ~ condition + (1|subject_nr) + (1|face_number), data=social_distance)

summary(m2)

m3 <- lmer(response ~ condition * Country + (1|subject_nr) + (1|face_number), data=social_distance)

summary(m3)

m4 <- lmer(response ~ condition * Country + condition * Political_preference + (1|subject_nr) + (1|face_number), data=social_distance)

summary(m4)

m5 <- lmer(response ~ condition * Country + condition * Political_preference + condition * Sequence + (1|subject_nr) + (1|face_number), data=social_distance)

summary(m5)

m6 <- lmer(response ~ condition * Country + condition * Political_preference + condition * Sequence + Condition * Disgust.c + (1|subject_nr) + (1|face_number), data=social_distance)

summary(m6)

m7 <- lmer(response ~ condition * Country + condition * Political_preference + condition * Sequence + condition * Disgust.c + condition * Social_Anxiety.c + (1|subject_nr) + (1|face_number), data=social_distance)

summary(m7)

m8 <- lmer(response ~ condition * Country + condition * Political_preference + condition * Sequence + condition * Disgust.c + condition * Social_Anxiety.c + condition * Trust1.c + condition * Trustworthiness6.c + (1|subject_nr) + (1|face_number), data=social_distance)

summary(m8)

m9 <- lmer(response ~ condition * Country + condition * Political_preference + condition * Sequence + condition * Disgust.c + condition * Social_Anxiety.c + condition * Trust1.c + condition * Trustworthiness6.c + condition * Gender + (1|subject_nr) + (1|face_number), data=social_distance)

summary(m9)

# Introducing random slopes

m10 <- lmer(response ~ condition * Country + condition * Political_preference + condition * Sequence + condition * Disgust.c + condition * Social_Anxiety.c + condition * Trust1.c + condition * Trustworthiness6.c + condition * Gender (condition * Country |subject_nr) + (condition * Country |face_number), data=social_distance)

summary(m10)

# model selection

compare_performance(m1, m2, m3, m4, m5, m6, m7,m8,m9, m10)

plot(compare_performance(m1, m2, m3, m4, m5, m6, m7, m8, m9, m10, rank = TRUE))

check_model(m_selected)

confint(m_selected, oldNames = FALSE)

# Cooks's Distance #

infl.m <- influence(m_selected, group="subject_nr")

plot(infl.m,which="cook")

cd.mi <- cooks.distance(infl.m)

cd.mi[cd.mi > 4/n]

cd.mi[cd.mi > 4/(n-k-1),] # k = number of predictor variables

# Predicted means

(m.m <-lsmeans(m_selected, "condition", type="response"))

m.summary <- summary(m.m)

contrast(m.m, method = "trt.vs.ctrl", adjust="Bonferroni")

(m.m1 <-lsmeans(m_selected, "Country", type="response"))

m1.summary <- summary(m.m1)

contrast(m.m1, method = "trt.vs.ctrl", adjust="Bonferroni")

(m.m2 <-lsmeans(m_selected, "Political_preference", type="response"))

m2.summary <- summary(m.m2)

contrast(m.m2, method = "trt.vs.ctrl", adjust="Bonferroni")

# Social_distance Plot: condition (masked vs. unmasked faces)

p1 <- ggplot(data = m.summary, aes(x =condition, y =lsmean, ymin=asymp.LCL, ymax =asymp.UCL, colour=condition)) + geom_point(position = position_dodge(width = 0.2)) + geom_errorbar(position = position_dodge(width = 0.2), width = 0.01) + scale_colour_manual(values = c("darkblue", "black")) + theme_bw()

p1 <- p1 + scale_y_continuous(limits=c(3 , 5),oob = rescale_none)

p1 <- (gg_plot <- ggplotly(p1, originalData = TRUE)) #%>% layout(autosize = T)

p1

## B. Trustworthiness

contrasts(trustworthiness $condition) <- c(-0.5, 0.5)

mo1 <- lmer(response ~ 1 + (1|subject_nr) + (1|face_number), data= trustworthiness)

summary(mo1)

mo2 <- lmer(response ~ condition + (1|subject_nr) + (1|face_number), data= trustworthiness)

summary(mo2)

mo3 <- lmer(response ~ condition * Country + (1|subject_nr) + (1|face_number), data= trustworthiness)

summary(mo3)

mo4 <- lmer(response ~ condition * Country + condition * Political_preference + (1|subject_nr) + (1|face_number), data= trustworthiness)

summary(mo4)

mo5 <- lmer(response ~ condition * Country + condition * Political_preference + condition * Sequence + (1|subject_nr) + (1|face_number), data= trustworthiness)

summary(mo5)

mo6 <- lmer(response ~ condition * Country + condition * Political_preference + condition * Sequence + condition * Disgust.c + (1|subject_nr) + (1|face_number), data= trustworthiness)

summary(mo6)

mo7 <- lmer(response ~ condition * Country + condition * Political_preference + condition * Sequence + condition * Disgust.c + condition * Social_Anxiety.c + (1|subject_nr) + (1|face_number), data= trustworthiness)

summary(mo7)

mo8 <- lmer(response ~ condition * Country + condition * Political_preference + condition * Sequence + condition * Disgust.c + condition * Social_Anxiety.c + condition * Trust1.c + condition * Trustworthiness6.c + (1|subject_nr) + (1|face_number), data= trustworthiness)

summary(mo8)

mo9 <- lmer(response ~ condition * Country + condition * Political_preference + condition * Sequence + condition * Disgust.c + condition * Social_Distance.c + condition * Trust1.c + condition * Trustworthiness6.c + condition * Gender + (1|subject_nr) + (1|face_number), data= trustworthiness)

summary(mo9)

# Introducing random slopes

mo10 <- lmer(response ~ condition * Country + condition * Political_preference + condition * Sequence + condition * Disgust.c + condition * Social_Distance.c + condition * Trust1.c + condition * Trustworthiness6.c + condition * Gender (condition * Country |subject_nr) + (condition * Country |face_number), data= trustworthiness)

summary(mo10)

# model selection

compare_performance(mo1, mo2, mo3, mo4, mo5, mo6, mo7, mo8, mo9, mo10)

plot(compare_performance(mo1, mo2, mo3, mo4, mo5, mo6, mo7, mo8, mo9, mo10, rank = TRUE))

check_model(mo_selected)

confint(mo_selected, oldNames = FALSE)

# Cooks's Distance #

infl.mo <- influence(mo_selected, group="subject_nr")

plot(infl.mo,which="cook")

cd.mi <- cooks.distance(infl.mo)

cd.mi[cd.mi > 4/n]

cd.mi[cd.mi > 4/(n-k-1),] # k = number of predictor variables

# Predicted means

(m.mo <-lsmeans(mo_selected, "condition", type="response"))

mo.summary <- summary(m.mo)

contrast(m.mo, method = "trt.vs.ctrl", adjust="Bonferroni")

(m.mo1 <-lsmeans(mo_selected, "Country", type="response"))

mo1.summary <- summary(m.mo1)

contrast(m.mo1, method = "trt.vs.ctrl", adjust="Bonferroni")

(m.mo2 <-lsmeans(mo_selected, "Political_preference", type="response"))

mo2.summary <- summary(m.mo2)

contrast(m.mo2, method = "trt.vs.ctrl", adjust="Bonferroni")

# Trustworthiness Plot: condition

p2 <- ggplot(data = mo2.summary, aes(x =condition, y =lsmean, ymin=asymp.LCL, ymax =asymp.UCL, colour=condition)) + geom_point(position = position_dodge(width = 0.2)) + geom_errorbar(position = position_dodge(width = 0.2), width = 0.01) + scale_colour_manual(values = c("darkblue", "black")) + theme_bw()

p2 <- p2 + scale_y_continuous(limits=c(3 , 5),oob = rescale_none)

p2 <- (gg_plot <- ggplotly(p2, originalData = TRUE)) #%>% layout(autosize = T)

p2

## C. Sickness

contrasts(sickness$condition) <- c(-0.5, 0.5)

mod1 <- glmer(response ~ 1 + (1|subject_nr) + (1|face_number), data= sickness, family = binomial(link=logit),na.action = na.omit, control = glmerControl(optimizer = "bobyqa"))

summary(mod1)

mod2 <- glmer(response ~ condition + (1|subject_nr) + (1|face_number), data= sickness, family = binomial(link=logit),na.action = na.omit, control = glmerControl(optimizer = "bobyqa"))

summary(mod2)

mod3 <- glmer(response ~ condition * Country + (1|subject_nr) + (1|face_number), data= sickness, family = binomial(link=logit),na.action = na.omit, control = glmerControl(optimizer = "bobyqa"))

summary(mod3)

mod4 <- glmer(response ~ condition * Country + condition * Political_preference + (1|subject_nr) + (1|face_number), data= sickness, family = binomial(link=logit),na.action = na.omit, control = glmerControl(optimizer = "bobyqa"))

summary(mod4)

mod5 <- glmer(response ~ condition * Country + condition * Political_preference + condition * Sequence + (1|subject_nr) + (1|face_number), data= sickness, family = binomial(link=logit),na.action = na.omit, control = glmerControl(optimizer = "bobyqa"))

summary(mod5)

mod6 <- glmer(response ~ condition * Country + condition * Political_preference + condition * Sequence + condition * Disgust.c + (1|subject_nr) + (1|face_number), data= sickness, family = binomial(link=logit),na.action = na.omit, control = glmerControl(optimizer = "bobyqa"))

summary(mod6)

mod7 <- glmer(response ~ condition * Country + condition * Political_preference + condition * Sequence + condition * Disgust.c + condition * Social_Anxiety.c + (1|subject_nr) + (1|face_number), data= sickness, family = binomial(link=logit),na.action = na.omit, control = glmerControl(optimizer = "bobyqa"))

summary(mod7)

mod8 <- glmer(response ~ condition * Country + condition * Political_preference + condition * Sequence + condition * Disgust.c + condition * Social_Anxiety.c + condition * Trust1.c + condition * Trustworthiness6.c + (1|subject_nr) + (1|face_number), data= sickness, family = binomial(link=logit),na.action = na.omit, control = glmerControl(optimizer = "bobyqa"))

summary(mod8)

mod9 <- glmer(response ~ condition * Country + condition * Political_preference + condition * Sequence + condition * Disgust.c + condition * Social_Anxiety.c + condition * Trust1.c + condition * Trustworthiness6.c + condition * Gender + (1|subject_nr) + (1|face_number), data= sickness, family = binomial(link=logit),na.action = na.omit, control = glmerControl(optimizer = "bobyqa"))

summary(mod9)

# Introducing random slopes

mod10 <- glmer(response ~ condition * Country + condition * Political_preference + condition * Sequence + condition * Disgust.c + condition * Social_Anxiety.c + condition * Trust1.c + condition * Trustworthiness6.c + condition * Gender + (condition * Country |subject_nr) + (condition * Country |face_number), data= sickness, family = binomial(link=logit),na.action = na.omit, control = glmerControl(optimizer = "bobyqa"))

summary(mod10)

# model selection

compare_performance(mod1, mod2, mod3, mod4, mod5, mod6, mod7, mod8, mod9, mod10)

plot(compare_performance(mod1, mod2, mod3, mod4, mod5, mod6, mod7, mod8,mod9, mod10, rank = TRUE))

check_model(mod_selected)

confint(mod_selected, oldNames = FALSE)

# Cooks's Distance #

infl.mod <- influence(mod_selected, group="subject_nr")

plot(infl.mod,which="cook")

cd.mi <- cooks.distance(infl.mod)

cd.mi[cd.mi > 4/n]

cd.mi[cd.mi > 4/(n-k-1),] # k = number of predictor variables

# Predicted means

(m.mod <-lsmeans(mod_selected, "condition", type="response"))

mod.summary <- summary(m.mod)

contrast(m.mod, method = "trt.vs.ctrl", adjust="Bonferroni")

(m.mod1 <-lsmeans(mod_selected, "Country", type="response"))

mod1.summary <- summary(m.mod1)

contrast(m.mod1, method = "trt.vs.ctrl", adjust="Bonferroni")

(m.mod2 <-lsmeans(mod_selected, "Political_preference", type="response"))

mod2.summary <- summary(m.mod2)

contrast(m.mod2, method = "trt.vs.ctrl", adjust="Bonferroni")

# Sickness Plot: condition

p3 <- ggplot(data = mod.summary, aes(x =condition, y =lsmean, ymin=asymp.LCL, ymax =asymp.UCL, colour=condition)) + geom_point(position = position_dodge(width = 0.2)) + geom_errorbar(position = position_dodge(width = 0.2), width = 0.01) + scale_colour_manual(values = c("darkblue", "black")) + theme_bw()

p3 <- p3 + scale_y_continuous(limits=c(0 , 0.6),oob = rescale_none)

p3 <- (gg_plot <- ggplotly(p3, originalData = TRUE)) #%>% layout(autosize = T)

p3

###### Implicit O-VAAST ######

# Loading packages

if (!require("pacman")) install.packages("pacman") pacman::p_load("plyr","dplyr","gdata","reshape","car","tidyr","magrittr", "data.table", "ggplot2","readr","stringr","psych","GPArotation")

# implicit dataset #

data <- read.csv("O_VAAST.csv", sep=",")

DF <- data

# Defining the "Subject", "Stimuli", "Movement" and "Category" as factors

to_factor <- c("participant","stim_number","Key","target")

DF[, to_factor] <- lapply(DF[, to_factor], factor)

## Cleaning dataset and Data exclusion

DF <- subset (DF, Block=="BlkMaskApproach" | Block=="BlkMaskAvoid")

# Average response time

# Calculating the average response time per participant

DFRTm <- data.frame (DF$Response_Time, DF$participant)

DFRTm <- aggregate (DFRTm[, 1], list(DFRTm$DF.participant), mean)

DFRTm <- rename.vars (DFRTm, c("Group.1","x"), c("participant","RT_Mean"))

arrange(DFRTm,RT_Mean)

# Incorrect trials

# Calculating the rate of incorrect trials per participant

DFACC <- data.frame (DF$Correct_Response, DF$participant)

DFACC <- aggregate (DFACC[, 1], list(DFACC$DF.participant), sum)

DFACC <- rename.vars (DFACC, c("Group.1","x"), c("participant","ACC_Sum"))

DFACC$ACC_rate <- (DFACC$ACC_Sum * 100) /20

arrange(DFACC,ACC_rate)

# Rate of participants having less than 60% of correct trials

(nrow(DFACC[DFACC$ACC_rate < 60, ]) / nrow(DFACC) )

# Removing participants having less than 60% of correct trials

DF <- merge (DF, DFACC, by.x.=c(participant), all=TRUE)

DF<- subset(DF, ACC_rate>60)

# Overall rate of incorrect trials

1 - (nrow(DF[DF$Correct_Response == 1, ]) / nrow(DF) )

# Removing incorrect trials.

DF_correct <- DF[DF$Correct_Response == 1, ]

# RT Cutoff # % of exclusion should be around 2%

# define RT filters (minimum and maximum).

min <- 200

max <- 2500

# Rate of exclusion if we keep only the RTs above min and below max

1 - (nrow(DF_correct[DF_correct$Response_Time > min & DF_correct$Response_Time < max, ]) / nrow(DF_correct) )

# We remove RTs above min and below max

DF_correct <- DF_correct[DF_correct$Response_Time > min & DF_correct$Response_Time < max, ]

## Model exploration: approach and avoidance RT

# Subset by action: approach or avoid

approach <- DF_correct[which(DF_correct $action == "approach"),]

avoid <- DF_correct [which(DF_correct $action == "avoid"),]

library(lmerTest)

## D. Approach model

fitA.lmer <- lmer(Response_Time ~ target * Country * Political_preference + (target * Country| participant) + (target * Country| stim_number), data = approach)

summary(fitA.lmer)

# Block moderation

fitA.lmer <- lmer(Response_Time ~ target * Country * Political_preference + target * Block_code +

(target * Country | participant) +

(target * Country | stim_number), data = approach)

summary(fitA.lmer)

# Predicted means

(m1 <-lsmeans(fitA.lmer, “target”, type="response"))

m1.summary <- summary(m1)

contrast(m1, method = "trt.vs.ctrl", adjust="Bonferroni")

(m2 <-lsmeans(fitA.lmer, “Country”, type="response"))

m2.summary <- summary(m2)

contrast(m2, method = "trt.vs.ctrl", adjust="Bonferroni")

(m3 <-lsmeans(fitA.lmer, “Political_preference”, type="response"))

m3.summary <- summary(m3)

contrast(m3, method = "trt.vs.ctrl", adjust="Bonferroni")

(m4 <-lsmeans(fitA.lmer, “target”, “Country”, “Political_preference”, type="response"))

m4.summary <- summary(m4)

contrast(m4, method = "trt.vs.ctrl", adjust="Bonferroni")

## E. Avoid model

fitB.lmer <- lmer(Response_Time ~ target * Country * Political_preference + (target * Country| participant) + (target * Country| stim_number), data = avoid)

summary(fitB.lmer)

# Block moderation

fitB.lmer <- lmer(Response_Time ~ target * Country * Political_preference + target * Block_code +

(target * Country| participant) +

(target * Country| stim_number), data = avoid)

summary(fitB.lmer)

# Predicted means

(m5 <-lsmeans(fitB.lmer, “target”, type="response"))

m5.summary <- summary(m5)

contrast(m5, method = "trt.vs.ctrl", adjust="Bonferroni")

(m6 <-lsmeans(fitB.lmer, “Country”, type="response"))

m6.summary <- summary(m6)

contrast(m6, method = "trt.vs.ctrl", adjust="Bonferroni")

(m7 <-lsmeans(fitB.lmer, “Political_preference”, type="response"))

m7.summary <- summary(m7)

contrast(m7, method = "trt.vs.ctrl", adjust="Bonferroni")

(m8 <-lsmeans(fitB.lmer, “target”, “Country”, “Political_preference”, type="response"))

m8.summary <- summary(m8)

contrast(m8, method = "trt.vs.ctrl", adjust="Bonferroni")

## References

Aguinis, H., Gottfredson, R. K., & Culpepper, S. A. (2013). Best-practice recommendations for estimating cross-level interaction effects using multilevel modeling. *Journal of Management,* *39*, 1490–1528. <https://doi.org/10.1177/0149206313478188>

American Psychiatric Association. (2013). *Diagnostic and statistical manual of mental disorders (DSM-5)*. American Psychiatric Publishing.

Baayen, R. H., Davidson, D. J., & Bates, D. M. (2008). Mixed-effects modeling with crossed random effects for subjects and items. *Journal of Memory and Language,* *59*, 390–412. <https://doi.org/10.1016/j.jml.2007.12.005>

Boelen, P. A., & Reijntjes, A. (2009). Intolerance of uncertainty and social anxiety. *Journal of Anxiety Disorders, 23*, 130–135. <https://doi.org/10.1016/j.janxdis.2008.04.007>

Corradi, G., Rosselló-Mir, J., Vañó, J., Chuquichambi, E. G., Bertamini, M., & Munar, E. (2018). The effects of presentation time on preference for curvature of real objects and meaningless novel patterns. *British Journal of Psychology,* *110*, 670–685. <https://doi.org/10.1111/bjop.12367>

Giner-Sorolla, R. (2018). *Powering your interaction*. <https://approachingblog.wordpress.com/2018/01/24/powering-your-interaction-2/>

Green, P., & MacLeod, C. J. (2016). SIMR: An R package for power analysis of generalized linear mixed models by simulation. *Methods in Ecology and Evolution,* *7*, 493–498.
<https://doi.org/10.1111/2041-210X.12504>

Judd, C. M., Westfall, J., & Kenny, D. A. (2017). Experiments with more than one random factor: Designs, analytic models, and statistical power. *Annual Review of Psychology,* *68*, 601–625. <https://doi.org/10.1146/annurev-psych-122414-033702>

Keselman, H. J., Algina, J., Kowalchuk, R. K., & Wolfinger, R. D. (1999). The analysis of repeated measurements: A comparison of mixed-model Satterthwaite f tests and a nonpooled adjusted degrees of freedom multivariate test. *Communications in Statistics – Theory and Methods,* *28*, 2967*–*2999. <https://doi.org/10.1080/03610929908832460>

Kuckertz, J. M., Strege, M. V., & Amir, N. (2017). Intolerance for approach of ambiguity in social anxiety disorder. *Cognition and Emotion,* *31*, 747–754. <https://doi.org/10.1080/02699931.2016.1145105>

Lange, W. G., Allart, E., Keijsers, G. P., Rinck, M., & Becker, E. S. (2012). A neutral face is not neutral even if you have not seen it: Social anxiety disorder and affective priming with facial expressions. *Cognitive Behaviour Therapy,* *41*, 108–118. <https://doi.org/10.1080/16506073.2012.666563>

Meteyard, L., & Davies, R. A. (2020). Best practice guidance for linear mixed-effects models in psychological science*. Journal of Memory and Language,* *112*, 104092. <https://doi.org/10.1016/j.jml.2020.104092>

Olivera-La Rosa, A., Chuquichambi, E. G., & Ingram, G. P. D. (2020). Keep your (social) distance: Pathogen concerns and social perception in the time of COVID-19. *Personality and Individual Differences,* *166*, 110200. <https://doi.org/10.1016/j.paid.2020.110200>

Sui, J., & Humphreys, G. W. (2013). The boundaries of self face perception: Response time distributions, perceptual categories, and decision weighting. *Visual Cognition,* *21*, 415–445. <https://doi.org/10.1080/13506285.2013.800621>

1. <https://osf.io/3bpdw/?view_only=fc1bfbfab1df48b6ac247d5d5e581542> [↑](#footnote-ref-1)
